# Coastal gradients and human disturbance shape bacterial and fungal rhizosphere microbiomes of *Heliotropium arboreum* in Hainan, China

**DOI:** 10.3389/fmicb.2026.1774048

**Published:** 2026-02-02

**Authors:** Xiaofeng Zhang, Linhua Sha, Youzhuan Mai, Jianhui Xu, Mir Muhammad Nizamani, Fazhi Fang

**Affiliations:** 1 Hainan Academy of Forestry (Hainan Academy of Mangrove), Haikou, China; 2 Key Laboratory of Tropical Forestry Resources Monitoring and Application of Hainan Province, Haikou, China; 3 Innovation Platform for Academicians of Hainan Province, Haikou, China; 4 Guangdong Provincial Key Laboratory of Marine Disaster Prediction and Prevention, Institute of Marine Sciences, Shantou University, Shantou, China

**Keywords:** alpha diversity, environmental filtering, functional guilds, oligotrophic soils, salinity gradient

## Abstract

Coastal ecosystems in Hainan exhibit steep sea–land gradients in salinity and nutrient availability, yet the rhizosphere microbiome of the pioneer shrub *Heliotropium arboreum* remains poorly understood. We investigated bacterial and fungal communities across seven coastal sites using replicated transects from seaward to shrub-belt to inland zones, and linked community patterns to soil physicochemical properties and human disturbance. Bacterial communities consistently showed higher richness, evenness, and compositional stability than fungal communities. Alpha diversity increased from seaward to inland zones for both groups, with a stronger gradient in fungi. Community composition was dominated by Proteobacteria and Planctomycetota in bacteria and Ascomycota in fungi, with distinct dominant genera across zones and sites. *β*-diversity analyses revealed clear differentiation of microbial communities among zones and locations, with fungi showing stronger turnover and site separation than bacteria, indicating higher sensitivity to environmental filtering and disturbance. Redundancy analysis indicated that fungal communities were primarily structured by available potassium, total nitrogen, and soil organic carbon, whereas bacterial communities were most strongly associated with soil pH (7.468–9.613 across sites) and nitrate concentrations. Functional profiling suggested complementary roles in decomposition and nitrogen cycling, and human-disturbed sites showed higher predicted pathogenic potential. Overall, *H. arboreum* hosts an environmentally filtered rhizosphere microbiome shaped jointly by coastal gradients and disturbance, with fungi responding more strongly than bacteria to spatial and environmental variation.

## 1 Introduction

Coastal ecosystems form dynamic transition zones between land and sea, where steep gradients in salinity, nutrient supply, and soil texture strongly influence the assembly of plant and microbial communities (Turner and Laliberté, 2015; Rath et al., 2019). Vegetation in these environments is typically stress tolerant, and interactions with rhizosphere microbes are pivotal for ecosystem stability, nutrient cycling, and plant persistence under fluctuating abiotic conditions (Liu et al., 2019). *Heliotropium arboreum*, a coastal shrub widely distributed along the islands and shorelines of Hainan Province in the South China, functions as a pioneer species in coral-sand and reef-derived soils. It often forms distinct banded vegetation patterns and establishes in substrates that are both saline and oligotrophic. Such persistence in nutrient-poor, high-stress habitats implies that *H. arboreum* may depend heavily on its rhizosphere microbiome for nutrient acquisition and stress mitigation. Recent surveys across multiple Hainan islands indicate that the rhizosphere bacterial community associated with *H. arboreum* is dominated by Proteobacteria and Planctomycetota, whereas fungal communities are enriched in Ascomycota and Basidiomycota. Environmental nutrient status, especially nitrogen, phosphorus, and potassium, appears to be a major driver of microbial assembly. Fungal taxa such as *Preussia* and *Metacordyceps* show strong positive associations with nutrient-enriched microsites, while bacterial diversity tends to be higher in less disturbed locations (Zhang et al., 2025). Despite the ecological importance of *H. arboreum*, the structure, functional potential, and environmental determinants of its soil microbiome remain insufficiently resolved. In contrast to mangrove and seagrass microbiomes, which have been intensively studied in Hainan’s coastal systems (Deng et al., 2024), microbial communities linked to *H. arboreum* represent a comparatively neglected component of tropical coastal ecology. Understanding how this shrub persists and stabilizes coastal substrates therefore requires a broader consideration of rhizosphere ecology and plant–microbe mutualisms.

The rhizosphere is one of the most dynamic and complex microbial habitats on Earth and acts as a central hub for ecosystem productivity and nutrient cycling. Interactions among roots, microbes, and the abiotic environment drive plant productivity, biogeochemical cycling, soil fertility, and stress tolerance (Mommer et al., 2016). Within this zone, beneficial symbioses such as plant growth–promoting bacteria and mycorrhizal fungi enhance nutrient uptake, suppress pathogens, and improve tolerance to drought, salinity, and heavy metals (Hol et al., 2014; Barra Caracciolo and Terenzi, 2021). Plants are not passive hosts in these relationships: through tailored root exudates they actively “engineer” microbial communities, selectively recruiting partners that align with their nutrient and stress needs (Ahkami et al., 2017; Brunel et al., 2020).

In the *H. arboreum* rhizosphere, which often develops under arid, oligotrophic, or saline soil conditions, the microbial community is typically enriched with *Bacillus*, *Paenibacillus*, and *Pseudomonas* species known for their phosphate solubilization, nitrogen fixation, and phytohormone (IAA and siderophore) production, promoting plant growth and drought tolerance (da Cunha et al., 2024). Studies of similar xerophytic rhizospheres reveal that deterministic selection processes—driven by plant root exudates and environmental constraints—favor keystone taxa that maintain nutrient cycling and microbial network stability under stress (López-Lozano et al., 2020). These microbial assemblages contribute to essential ecosystem services such as nitrogen fixation, phosphorus mobilization, and organic matter decomposition, helping *H. arboreum* and related desert species persist in poor soils (Zhou et al., 2025).

This tight functional integration supports the holobiont perspective, where plants and their microbiomes co-evolve as a single ecological unit contributing to adaptation, resilience, and biogeochemical stability (Brunel et al., 2020; Klein et al., 2022).

Research in coastal systems shows how strongly environmental context shapes these plant–microbe assemblies. Microbial diversity and function in coastal rhizospheres are structured along gradients of salinity, nutrient availability, sediment type, and metal concentrations, with Proteobacteria frequently dominating (Liu et al., 2022; Thomson et al., 2022). Rising salinity acts as a primary environmental filter that simplifies microbial networks and reduces alpha diversity, while halophytic plants counter this stress by recruiting salt-tolerant taxa that help maintain nitrogen cycling and organic matter turnover (Li et al., 2024; Qiu et al., 2021). Anthropogenic disturbances such as land-use change, pollution, and agriculture further erode microbial richness and disrupt nitrogen functional genes, weakening ecological stability; disturbed mangrove zones similarly show lower fungal diversity and more opportunistic taxa than undisturbed sites (Wu et al., 2023; Portalanza et al., 2025). Climate change amplifies these pressures, with warming, salinity intrusion, and nutrient enrichment shifting rhizosphere communities toward pathogen-dominated states and threatening long-evolved symbioses essential for coastal resilience (Hacquard et al., 2022). Across coastal succession, pioneer plants like *Suaeda glauca* and *Phragmites communis* recruit distinct microbial partners depending on successional stage and nutrient status, illustrating how plant–microbe co-selection preserves ecosystem functioning along persistent stress gradients (Dai et al., 2025). These patterns suggest that the success of *H. arboreum* in Hainan’s saline, oligotrophic shorelines may likewise be mediated by microbial partners tuned to local environmental constraints. Recent analyses of *H. arboreum* rhizosphere communities in Hainan’s coastal ecosystems reveal that the bacterial assemblage is dominated by *Proteobacteria*, *Planctomycetota*, and *Acidobacteriota*, with nitrogen- and phosphorus-associated taxa showing strong positive correlations with nutrient availability (Zhang et al., 2025). These microbial consortia appear to confer resilience under salinity and nutrient stress, paralleling patterns observed in other coastal halophytes where microbial recruitment enhances nutrient cycling and osmotic balance (Dai et al., 2025; Tugbaeva et al., 2025). Thus, the ecological success of *H. arboreum* on Hainan’s coastal soils likely reflects a co-adapted plant–microbe strategy for survival in saline, nutrient-poor conditions.

Although amplicon sequence variant (ASV)–based approaches provide higher taxonomic resolution by distinguishing single-nucleotide differences among sequences, operational taxonomic unit (OTU) clustering at a 97% similarity threshold remains widely applied in ecological studies that focus on community-level patterns rather than strain-level variation. In this study, our primary objective was to evaluate broad spatial gradients, diversity patterns, and environmental drivers of soil microbial communities associated with *H. arboreum* across contrasting coastal habitats. OTU-based clustering offers a robust and comparable framework for such large-scale ecological analyses, particularly when consistency with previous soil and coastal microbiome studies is required. Moreover, OTU-based methods facilitate direct comparison with earlier long-read sequencing studies conducted in similar environments, thereby strengthening the ecological interpretation of the results. Given this knowledge gap, the distribution of *H. arboreum* provides an ideal model for exploring how plant–soil–microbe interactions stabilize vegetation in transitional coastal zones. This study investigates the hypothesis that the ecological stability of *H. arboreum* bands is linked to a specific and functionally tailored soil microbiome shaped by both natural environmental gradients and anthropogenic pressure. By combining high-throughput sequencing with detailed soil physicochemical analyses across seven coastal sites in Hainan. This research aims to (i) characterize soil physicochemical properties together with microbial diversity and composition in *H. arboreum* soils, (ii) identify key soil environmental factors (e.g., pH, nutrient availability, and organic matter) structuring these microbial communities, and (iii) assess how human disturbance modifies soil conditions and, in turn, microbial ecological function and resilience.

## 2 Materials and methods

### 2.1 Research site information and sampling design

After a preliminary field survey to confirm the presence and banded distribution of *H. arboreum* and to identify suitable sea–land transects, we established quadrats and collected soil samples at seven locations in Hainan (Supplementary Table S1). Each site is centered on the banded community of *H. arboreum*, and three parallel 5 m × 5 m quadrats are set at 10 m on both sides of the banded area. For example, in Zhaoshu Island (A), the quadrat near the seawater is recorded as Aa, the quadrat centered on *H. arboreum* community is recorded as Ab, and the quadrat on the other side of *H. arboreum* community (far from the ocean) is recorded as Ac. B stands for Yongxing Island, C stands for North Island, L stands for the coast of Lingao County, W stands for Wuzhizhou Island, M stands for EMan and D stands for Danzhou. M and D are on the same coastline, only because they are separated from each other, so they are sampled separately. Among the seven research sites, the environment of three banded areas (quadrats) in each site is different, from close to seawater to far from seawater. Locations A, B and C are located in Sansha City, Hainan Province, and are part of the Xisha Islands in the South China Sea. There are sporadic *Sesuvium portulacastrum* growth (Aa, Ba, Ca) on the white beach (coral sand) near the sea. Research sites L, M and D are all black reefs near the sea, and no plants grow (La, Ma, Da). Although there is also a white beach in the area near the sea in research sites W, there are no plants (Wa). Among the seven research sites, in the strip-shaped quadrat of *H. arboreum*, except *H. arboreum*, the main species are *Scaevola taccada* and *Opuntia dillenii* (Ab, Bb, Cb, Lb, Mb, Db, Wb). Among the seven sites (Ac, Bc, Cc, Lc, Mc, Dc, Wc) far away from the ocean, the plant species are more abundant, and the different species are different due to human interference, but there is no *H. arboreum* growing in this area (Supplementary Table S1). The *H. arboreum* just grows there, neither too close to the ocean (because there are plants like amaranth closer to the ocean) nor far away from the ocean (there are other rich plants that can grow farther than them).

### 2.2 Soil sample collection and physical and chemical properties detection

Three quadrats were established in each sampling strip, and soil samples were collected via the five-point sampling method for homogenization into composite samples. Specifically, five soil cores (each with a depth of 20 cm) were taken from the five sampling points within a single quadrat and thoroughly mixed to form one composite sample. As a result, three composite samples were yielded per sampling strip, and nine composite samples were obtained for each research plot. Across all seven research sites included in this study, a total of 63 composite soil samples were collected. All soil samples were immediately placed in sterile self-sealing bags, labeled with corresponding sample numbers and collection dates, and stored at low temperature for subsequent processing. Take it back to the laboratory and further process the samples. After removing large impurities, the soil was screened with a 2 mm microporous screen, and the treated soil was divided into two parts: 5 g of each sample was placed into a sterile centrifuge tube and submitted to a sequencing company for molecular sequencing, while the remaining portion (approximately 300 g) was air-dried naturally and stored in a sterile self-sealing bag for subsequent determination of soil physical and chemical properties. Physical and chemical properties of soil (pH value, total nitrogen (TN), total potassium (TK), total phosphorus (TP), ammonium nitrogen (NH_4_^+^), available phosphorus (AP), available potassium (AK), nitrate nitrogen (NO_3_^−^) and organic matter) were determined by the previously reported method (Supplementary Table S2) (Fang et al., 2023).

### 2.3 DNA extraction and PCR amplification of soil microorganisms

Total genomic DNA was extracted from the soil samples using the PowerSoil DNA Isolation Kit (MO BIO Laboratories, San Diego, CA, USA), following the manufacturer’s protocol precisely. The quality and integrity of the extracted DNA were assessed through 1% agarose gel electrophoresis, while its concentration and purity were measured with a NanoDrop One spectrophotometer. After quality evaluation, all DNA extracts were combined for subsequent PCR amplification and sequencing analyses. For fungal community analysis, full-length ITS primers were used: ITS5-1737F (5′-GGAAGTAAAAGTCGTAACAAGG-3′) and ITS2-2043R (5′-GCTGCGTTCTTCATCGATGC-3′). For bacterial communities, the V4–V5 region of the 16S rRNA gene was amplified using primers 515F (5′-barcode-GTGCCAGCMGCCGCGG-3′) and 907R (5′-CCGTCAATTCMTTTRAGTTT-3′). PCR amplification was performed under the following conditions: an initial denaturation at 95 °C for 5 min; 27 cycles of denaturation at 95 °C for 30 s, annealing at 55 °C for 30 s, and extension at 72 °C for 60 s; followed by a final extension at 72 °C for 10 min. The resulting PCR products were purified, quantified, and normalized before library construction. After library preparation and quality assessment, sequencing was carried out on the PacBio platform. All experimental steps, including DNA extraction, PCR amplification, and sequencing, were completed by Guangdong Meige Gene Technology Co., Ltd. During raw data processing, Trimmomatic was used to filter low-quality reads from the fastq files. The cleaned reads were then denoised using the unoise3 algorithm with default parameters. Following previously reported methodologies (Liu et al., 2020), chimeric sequences were removed, and the reads were assembled using FLASH software.

### 2.4 Bioinformatics and statistical analyses

Sequence data were processed using USEARCH (version 10.0) for OTU clustering at a 97% sequence similarity threshold (Quast et al., 2012). Operational Taxonomic Units (OTUs) with relative abundances below 0.005% of total reads were removed (Kõljalg et al., 2013). Taxonomic assignment of OTU representative sequences was conducted using the UNITE database (Release 8.0) and the SILVA database (Release 132) with a minimum similarity criterion of 0.8 (Kõljalg et al., 2005; Callahan et al., 2016). Functional guild classification of fungal taxa was performed using FUNGuild (Nguyen et al., 2016), while bacterial phenotypic traits were predicted using BugBase (Ward et al., 2017).

Statistical analyses were carried out in R (version 4.3.2). Alpha diversity metrics, including Chao1, Shannon, Simpson, and ACE, were calculated using USEARCH (alpha_div) and the corresponding R packages. Differences among groups were tested using one-way ANOVA, and false discovery rate (FDR) correction was applied for multiple comparisons. Community structure was evaluated using non-metric multidimensional scaling (NMDS) based on Bray–Curtis distances implemented in the vegan package in R. Relationships between microbial community composition and environmental variables were assessed using OTU abundance data and measured environmental parameters, and the results were visualized using heatmaps.

## 3 Results

### 3.1 Fungal and bacterial community composition and diversity patterns

The sequencing analysis confirmed consistently high data quality across all *H. arboreum* sites, with Q20 values exceeding 99% and Q30 values above 99%, indicating reliable read accuracy and depth for downstream analyses. A total of 5,255,082 effective fungal sequences and 5,308,025 effective bacterial sequences were obtained from the 63 soil samples, with a corresponding identification of 23,592 and 30,5,685 OTUs (Supplementary Tables S3, S4), respectively. Alpha diversity indices, including Chao1, ACE, and Shannon, demonstrated higher evenness among bacterial communities compared to fungi, reflecting a more stable and uniform bacterial structure across *H. arboreum* sampling sites (Supplementary Tables S5, S6). Furthermore, the environmental parameters summarized in Supplementary Tables S1, S2 revealed distinct site-specific variations in soil pH (7.468–9.613 across sites), soil organic carbon (0.760–62.958), and nutrient concentrations, including total nitrogen (0.074–4.291), total phosphorus (0.086–46.249), total potassium (0.031–3.925), available phosphorus (0.298–9.163), available potassium (7.810–608.470), NH_4_^+^ (2.196–11.649), and NO_3_^−^ (0.153–41.936), all of which were strongly correlated with the observed patterns of *H. arboreum* microbial diversity and community composition.

**Figure 1:**
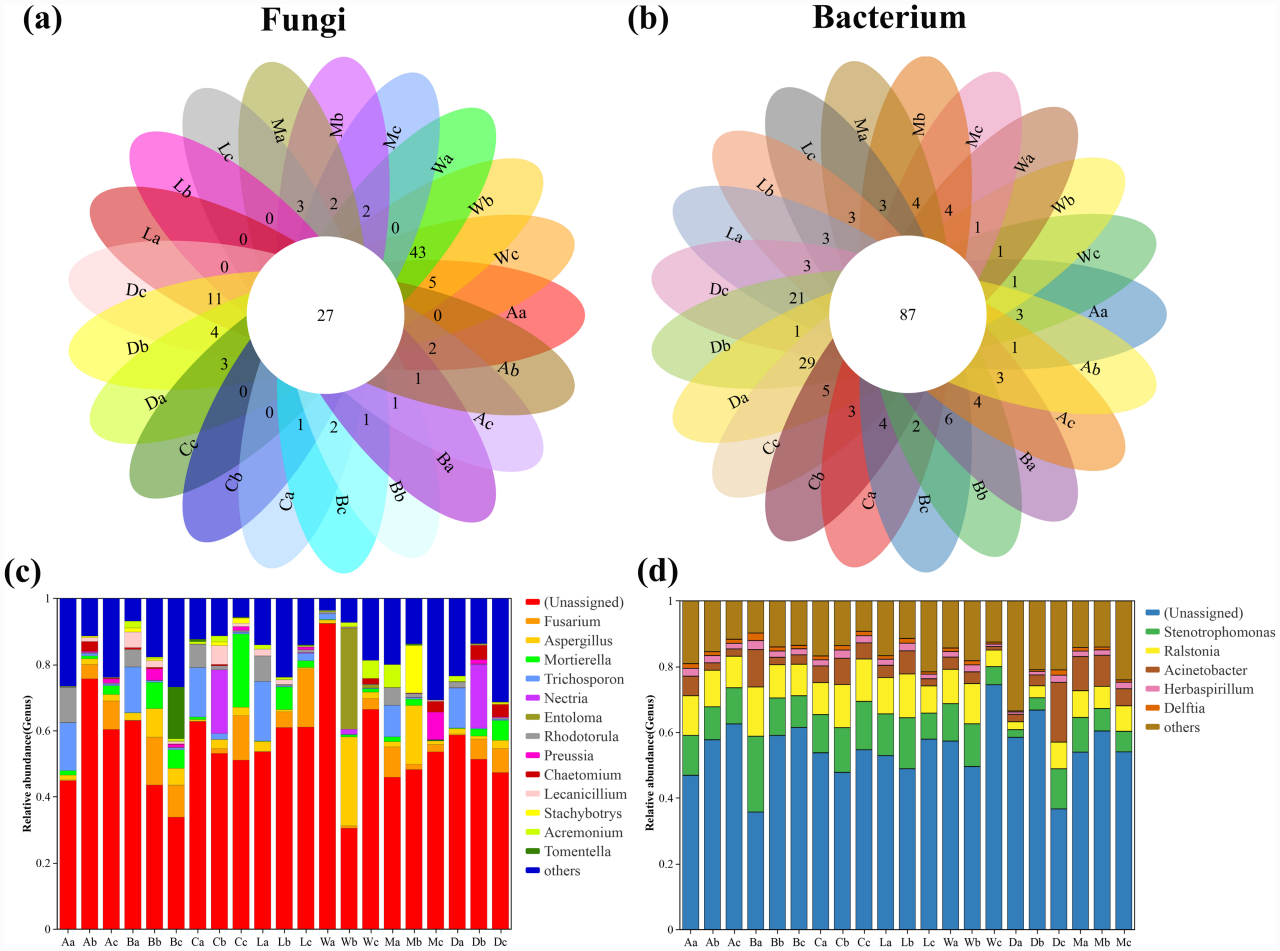
Shared and dominant taxa patterns of fungal and bacterial communities in *H. arboreum* rhizosphere soils across Hainan coastal sites. **(a)** Petal (flower) diagram of fungal OTUs/ASVs showing the number of unique taxa in each sample; the number in the center indicates core taxa shared by all samples. **(b)** Petal (flower) diagram of bacterial OTUs/ASVs (center = core taxa shared by all samples; petals = unique taxa). **(c)** Genus-level relative abundance of dominant fungal genera across samples (remaining genera merged as “Others”). **(d)** Genus-level relative abundance of dominant bacterial genera across samples (remaining genera merged as “Others”). *Sample codes are site + zone: a = seaward, b = shrub belt, c = inland.*

A total of 27 unique fungal species and 87 bacterial species were identified across the seven sampling sites associated with *H. arboreum*. Fungal communities exhibited marked spatial variation among the sites. Locations A, B and C are far away from the mainland, and there is little difference in fungal communities from near seawater (A) to far from the ocean (C), but locations D and W are obviously different, showing a high number of unique fungal taxa (Figure 1a). This pronounced distinctiveness at site W aligns with its status as a tourist island, where intensive human activities from Wb to Wc, including frequent foot traffic, shoreline recreation, and soil disturbance, can create microhabitats that support opportunistic or disturbance-tolerant fungal groups. Similarly, the uniqueness observed at Db and Dc may be linked to their proximity to the coastal highway, where cyclists and visitors often stop, resulting in localized trampling and nutrient inputs that alter soil fungal assemblages. In contrast, the bacterial community shows higher richness and uniformity, especially in Danzhou (D) and Lingao coastal (La) areas, which may reflect higher nutrient input and human interference (Figure 1b). In La, local residents frequently collect shellfish and dig in the intertidal zone; these repeated small-scale disturbances increase organic matter turnover and nutrient release, creating conditions that promote bacterial proliferation and favor taxa adapted to nutrient-enriched or frequently reworked soils. Dominant fungal genera included *Fusarium*, *Aspergillus*, *Mortierella*, and *Trichosporon*, all of which are known decomposers contributing to soil nutrient cycling within *H. arboreum* habitats. The results show that except Eman (M), the proportion of *Fusarium* from quadrat b to quadrat c is very high in the other six locations; In addition, except Wuzhizhou Island (W), *Trichosporon* accounts for a high proportion in the quadrat numbered a in the other six locations (Figure 1c). The reduced presence of *Trichosporon* at W may indicate sensitivity to the more intense human disturbance characteristic of this tourist site. The bacterial community was dominated by *Stenotrophomonas*, *Ralstonia*, and *Acinetobacter*, taxa commonly associated with nitrogen fixation and organic matter degradation in *H. arboreum*-associated soils. The abundance of unclassified bacteria highlights current gaps in microbial taxonomic databases (Figure 1d).

**Figure 2:**
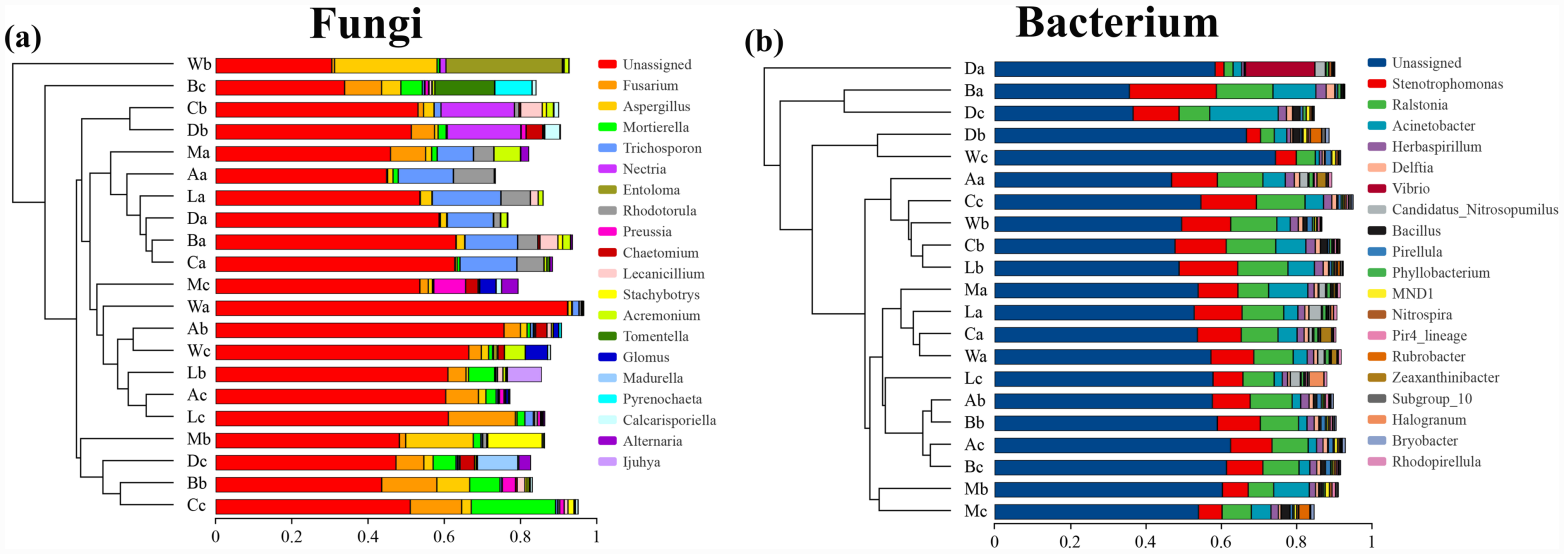
Hierarchical clustering and genus-level composition of fungal and bacterial communities in *H. arboreum* rhizosphere soils across coastal sites in Hainan. **(a)** Fungal communities: hierarchical clustering of samples (based on community dissimilarity) with stacked bars showing the relative abundance of dominant fungal genera. **(b)** Bacterial communities: hierarchical clustering of samples with stacked bars showing the relative abundance of dominant bacterial genera. *Sample codes are site + zone: a = seaward, b = shrub belt, c = inland.*

The distribution of fungal genera revealed a consistent dominance of *Fusarium*, followed by *Aspergillus*, *Mortierella*, *Trichosporon*, and *Preussia* (Figure 2a). These taxa contribute significantly to organic matter decomposition and soil health within *H. arboreum* ecosystems. The bacterial genera *Stenotrophomonas*, *Ralstonia*, *Acinetobacter*, *Herbaspirillum*, and *Vibrio* were prevalent across sites, emphasizing their ecological roles in nutrient cycling and nitrogen metabolism in *H. arboreum* rhizospheres (Figure 2b). A considerable proportion of sequences were categorized as “unclassified,” reflecting hidden microbial diversity requiring advanced taxonomic resolution.

### 3.2 Alpha and Beta diversity analysis

**Figure 3:**
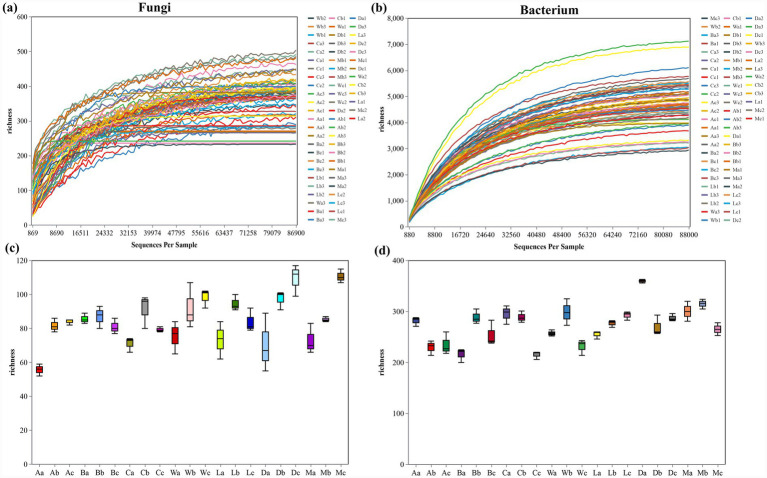
Rarefaction curves and alpha diversity of fungal and bacterial communities in *H. arboreum* rhizosphere soils: **(a)** Rarefaction curves (fungi); **(b)** rarefaction curves (bacteria); **(c)** fungal alpha diversity (richness) across samples; **(d)** bacterial alpha diversity (richness) across samples.

Rarefaction and alpha diversity analyses revealed significant differences in microbial richness across *H. arboreum* sites (Figure 3). For fungi, species richness increased with sequencing depth, reaching saturation, confirming adequate sampling coverage (Figure 3a). The highest fungal richness occurred at Wuzhizhou Island (W) and Beidao Island (C), while Zhaoshu Island (A) and Lingao coastal (L) exhibited lower diversity (Figure 3c). Bacterial richness was consistently higher, with operational taxonomic units (OTUs) reaching approximately 8,000 across *H. arboreum* samples (Figure 3b). Danzhou (D) and Eman (M) displayed the greatest bacterial richness, likely driven by elevated nutrient cycling and anthropogenic input (Figure 3d). These results indicate that bacterial communities associated with *H. arboreum* exhibit higher stability and adaptability than fungal communities under varying environmental conditions.

Microbial richness displayed a clear spatial pattern across the seven *H. arboreum* research sites, as shown by the rarefaction curves and alpha-diversity boxplots. In both fungi and bacteria, richness tended to be lowest in the quadrats closest to the sea (the “a” positions), where strong salt stress, coral sand substrates, or bare reef surfaces restrict microbial development. Richness generally increased in the central quadrats (the “b” positions), which correspond to the *H. arboreum* belt and therefore contain more root activity, litter input, and moderate soil moisture. The highest richness often occurred in the inland quadrats (the “c” positions), where plant diversity and soil organic matter were greater and environmental stress was lower. This offshore-to-inland gradient (a → b → c) was especially pronounced at Wuzhizhou Island (W), Beidao Island (C), Eman (M), and Danzhou (D). Although fungi and bacteria followed the same overall pattern, bacterial richness was consistently higher and exhibited greater stability across sites, reflecting stronger adaptability to varying coastal environmental conditions. This spatial trend, observed repeatedly across all seven regions, suggests that both microbial groups respond sensitively to ecological transitions from marine influence to inland vegetation zones (Figures 3c,d).

**Figure 4:**
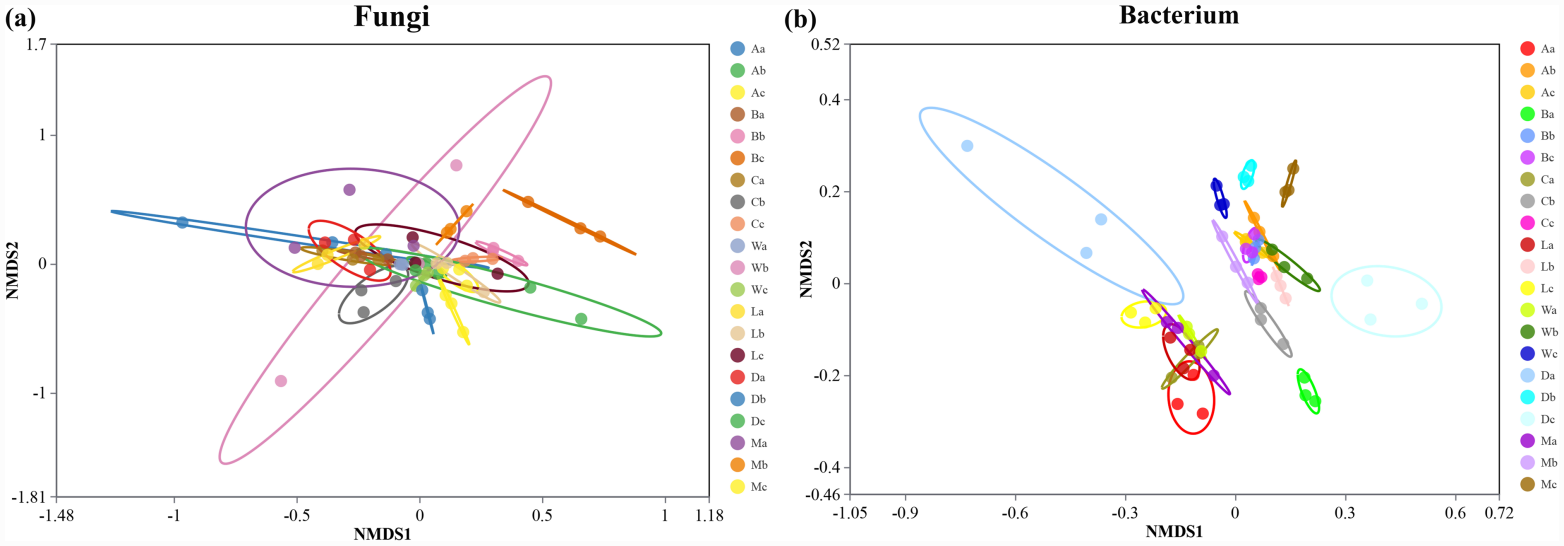
NMDS ordination of fungal and bacterial community structures in *H. arboreum* rhizosphere soils: **(a)** Fungal NMDS and **(b)** Bacterial NMDS. Points represent samples and are coloured by site/zone.

Non-metric Multidimensional Scaling (NMDS) plots demonstrated distinct clustering of fungal and bacterial communities among *H. arboreum* sites (Figures 4a,b). Fungal communities from Zhaoshu Island (A) and Lingao coastal (L) were closely related, whereas those from Wuzhizhou Island (W) and Danzhou coastal (D) showed distinct composition patterns (Figure 4a). Bacterial communities exhibited more homogeneous clustering, with Wuzhizhou (W) and Danzhou (D) again showing separation linked to environmental and anthropogenic gradients (Figure 4b). These results highlight the influence of ocean proximity and human activity on *H. arboreum* -associated microbial community differentiation.

**Figure 5:**
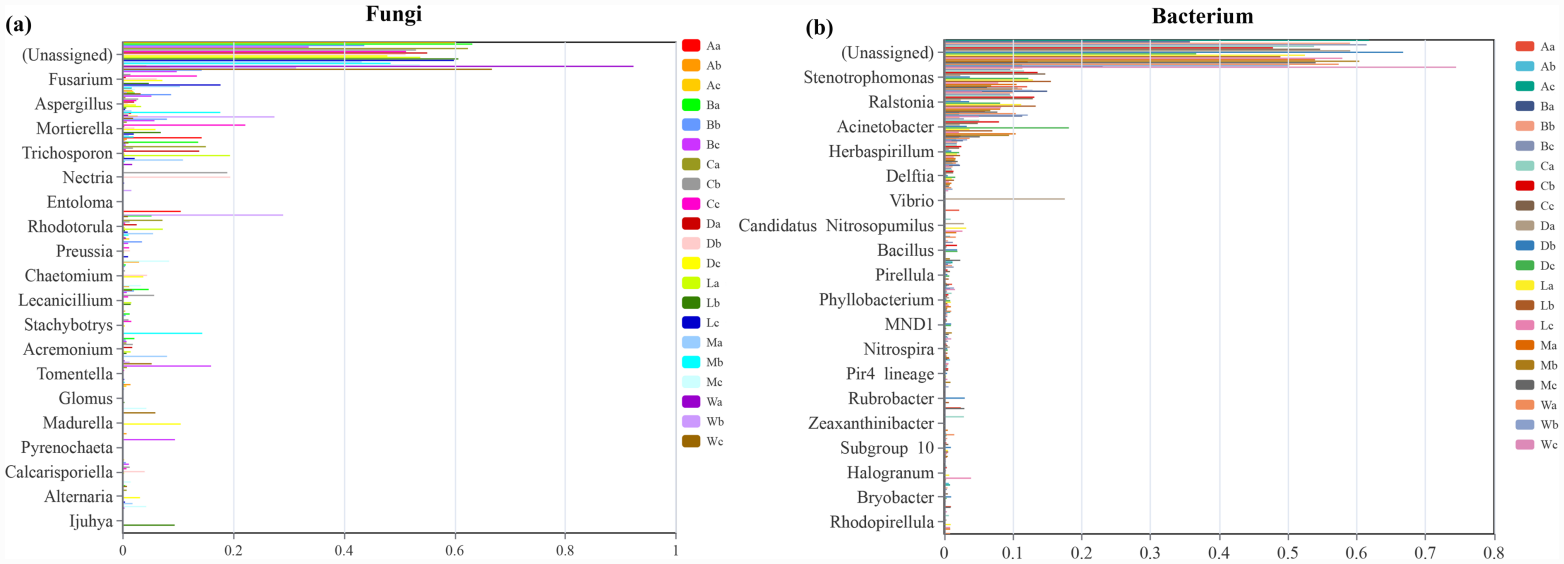
Multi-group comparison of dominant fungal and bacterial genera across coastal sites. **(a)** Relative abundance of dominant fungal genera. **(b)** Relative abundance of dominant bacterial genera. Samples are grouped by site and zone (a = seaward, b = shrub belt, c = inland).

Comparative abundance analyses revealed clear site-specific variations in dominant genera within *H. arboreum* soils. Fungal genera *Fusarium* and *Aspergillus* were most abundant in Zhaoshu (A) and Wuzhizhou (W), whereas *Mortierella* and *Stachybotrys* dominated in Danzhou (D) and Lingao (L) (Figure 5a). For bacteria, *Stenotrophomonas* and *Ralstonia* were more prevalent in Wuzhizhou (W) and Danzhou (D), while Zhaoshu (A) and Lingao (L) supported more balanced microbial compositions (Figure 5b). These differences underline the strong environmental filtering effect of local soil and climatic factors on *H. arboreum* microbial communities.

### 3.3 Environmental correlations (RDA and heatmap analyses)

**Figure 6:**
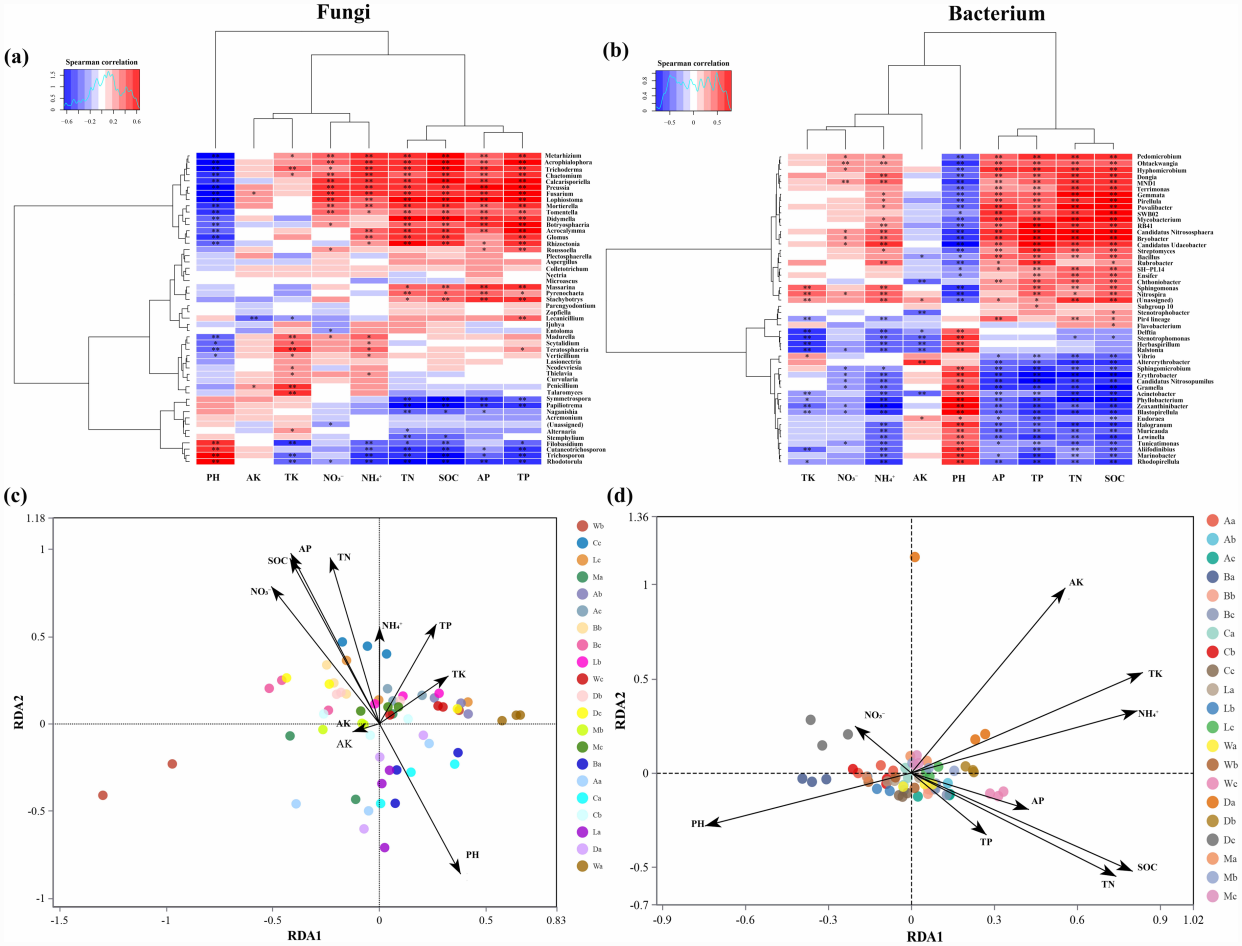
Relationships between soil physicochemical properties and dominant microbial genera in *H. arboreum* rhizosphere soils. **(a)** Spearman correlation heatmap for fungal genera vs. soil properties. **(b)** Spearman correlation heatmap for bacterial genera vs. soil properties. **(c)** RDA ordination of fungal communities constrained by soil variables. **(d)** RDA ordination of bacterial communities constrained by soil variables.

For fungi, available potassium (AK), total nitrogen (TN), and soil organic carbon (SOC) exerted the most pronounced effects, especially in Zhaoshu (A) and Wuzhizhou (W) (Figure 6a). In bacterial communities, nitrate (NO₃^−^) and pH were the principal drivers of variation, particularly in Danzhou (D) and Wuzhizhou (W). Heatmap correlations indicated positive associations between *Fusarium* and *Aspergillus* with SOC and TN, while *Stenotrophomonas* correlated strongly with NO₃^−^ (Figure 6b). Redundancy Analysis (RDA) plots revealed strong correlations between *H. arboreum* microbial community composition and soil parameters (Figures 6c,d). These findings emphasize the role of nutrient availability and soil chemistry in shaping *H. arboreum* -associated microbial assemblages.

### 3.4 Taxonomic and functional profiling

**Figure 7:**
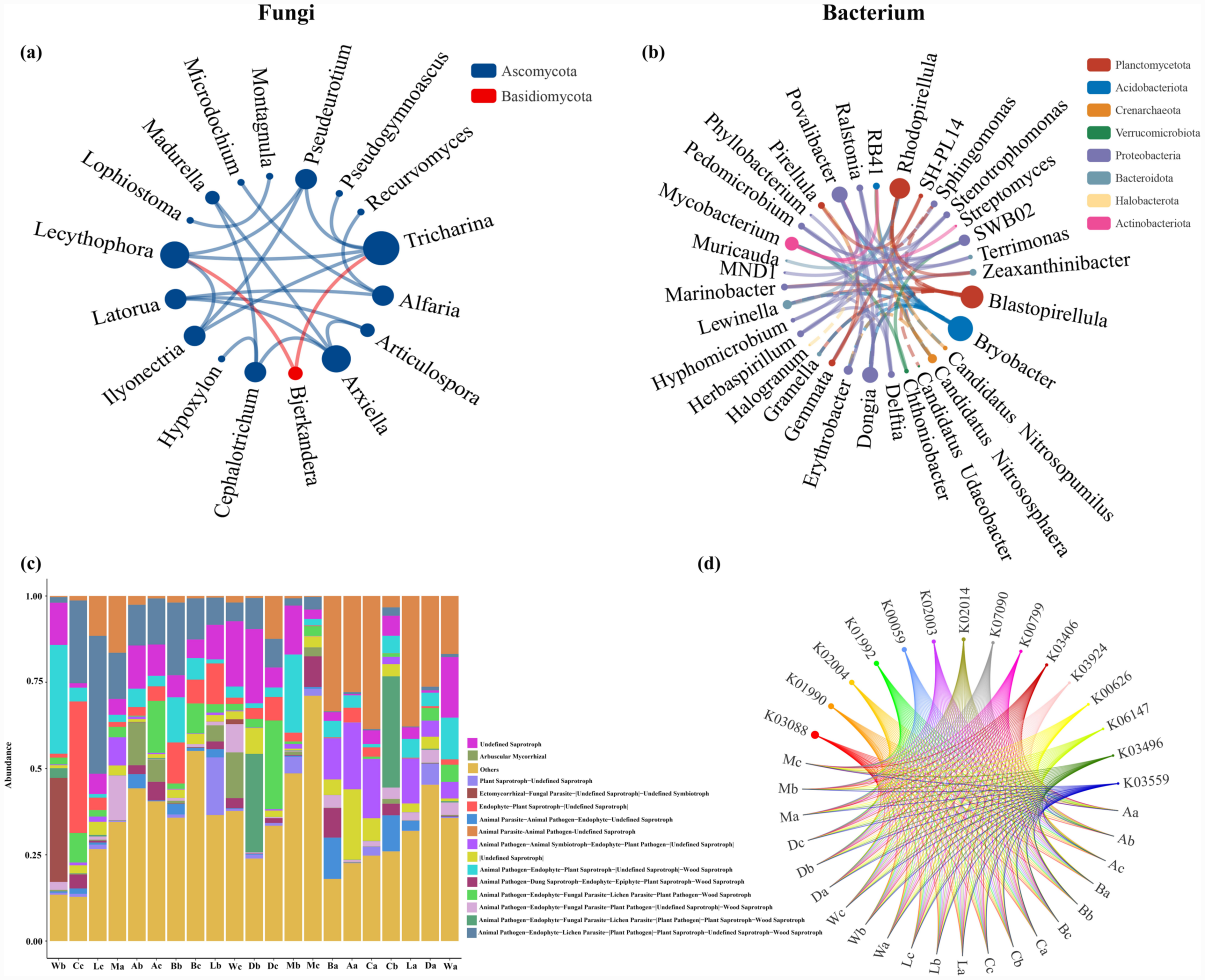
Taxonomic and functional profiling of the *H. arboreum* rhizosphere microbiome. **(a)** Fungal co-occurrence network of dominant genera; node colour indicates phylum (e.g., Ascomycota and Basidiomycota). **(b)** Bacterial co-occurrence network of dominant genera; node colour indicates phylum. **(c)** Predicted functional guild composition of fungal communities across samples (e.g., FUNGuild categories). **(d)** Chord diagram showing associations between sample groups and predicted functional features (KEGG orthologs; K numbers).

Network and functional analyses provided insights into the taxonomic structure and putative ecological roles of *H. arboreum*–associated microbiota (Figures 7a–d). Fungal taxa predominantly belonged to Ascomycota, with *Microdochium*, *Montagnula*, and *Lecythophora* as representative genera, while Basidiomycota members (*Bickerndera*, *Tricharina*) were less frequent (Figure 7a). Bacterial taxa were primarily within Proteobacteria, Planctomycetota, and Acidobacteria, with *Stenotrophomonas*, *Ralstonia*, and *Herbaspirillum* as key representatives (Figure 7b). Functional guilds and phenotypes were inferred using FUNGuild and BugBase, which assign functions based on curated reference databases and taxonomy-linked annotations rather than direct measurement of genes or activity. As a result, predictions can be constrained by database coverage, taxonomic resolution, and annotation uncertainty, and may be biased toward well-characterised taxa or environments; some taxa may also be assigned to broad or multiple guild categories. Therefore, functional differences should be interpreted as indicative trends rather than definitive evidence. Within these constraints, functional profiles suggested site-dependent differences, with Wuzhizhou (W) and Danzhou (D) harbouring higher proportions of predicted plant- and animal-associated pathogenic guilds/phenotypes, while other *H. arboreum* sites exhibited more saprophytic and decomposer-associated taxa (Figure 7c). Chord diagrams indicated multifunctional associations of dominant genera, with *Fusarium* linked to both decomposer- and pathogen-associated guilds, while *Stenotrophomonas* and *Ralstonia* were associated with predicted nitrogen cycling and organic matter–related functional categories in *H. arboreum* rhizospheres (Figure 7d).

## 4 Discussion

### 4.1 Diversity patterns and dominant microbial taxa (bacteria vs. fungi)

Across all *H. arboreum* sites, bacterial communities showed higher richness, evenness, and compositional stability than fungal communities. This consistent pattern, as supported by previous research, indicates that bacteria are better able to persist and reorganize under strong spatial heterogeneity and recurrent stress typical of coastal environments. Studies have shown that bacterial networks maintain higher stability and complexity under aridity and salinity, while fungal networks become less stable due to their narrower ecological niches and slower adaptive capacity (Chen et al., 2022). Similarly, bacterial dispersal across heterogeneous landscapes enhances resilience and ecological connectivity, whereas fungi tend to form site-specific communities strongly shaped by vegetation and nutrient availability (Schiro et al., 2022; Liu et al., 2023). Therefore, the higher bacterial diversity and stability observed in *H. arboreum* rhizospheres likely reflect bacteria’s broader metabolic flexibility, faster population turnover, and greater tolerance to coastal stress gradients, whereas fungal communities are more tightly filtered by local conditions and environmental variability.

The prevalence of bacterial genera such as *Stenotrophomonas*, *Ralstonia*, and *Acinetobacter* supports their central role in sustaining nutrient cycling in *H. arboreum* soils (Figures 1d, 2b). These taxa are frequently associated with nitrogen transformations and organic matter decomposition, processes that are especially critical in oligotrophic, saline substrates where plant growth depends on efficient microbial nutrient mobilization (Zhang et al., 2025). In contrast, fungal communities in the same habitats were less diverse and more variable among sites, with *Fusarium*, *Aspergillus*, and *Mortierella* dominating. The stronger site sensitivity of fungi is consistent with evidence that fungal assemblages often respond sharply to salinity stress, nutrient limitation, and disturbance because many lineages have slower growth rates and narrower realized niches than bacteria (Zhang et al., 2023). These results indicate a bacterial community that is broadly resilient across the coastal gradient and a fungal community whose structure is more tightly filtered by local conditions.

### 4.2 Unclassified reads and database gaps in coastal/tropical soils

A substantial fraction of bacterial reads remained unclassified in *H. arboreum* rhizosphere soils. This pattern highlights persistent gaps in reference databases for coastal and tropical environments, where many lineages are poorly represented by cultivated isolates or genome-resolved studies. Unclassified reads may therefore reflect true microbial novelty rather than analytical error. Similar “dark matter” fractions are repeatedly reported in tropical soils and marine-influenced sediments, where many taxa cannot be assigned reliably below high taxonomic ranks due to sparse reference coverage (Chan and Chong, 2014; Anthony et al., 2024). Even in well-studied, high-complexity soils, metagenomic and amplicon pipelines typically leave a large portion of sequences unannotated, indicating that incomplete databases remain a global bottleneck rather than a region-specific issue (Edwin et al., 2024). Reports from Subantarctic and coral-sediment microbiomes likewise show high proportions of unclassified OTUs, reinforcing the idea that phylogenetically distinct lineages are widespread across climatic zones (Guibert et al., 2016).

Recent initiatives, including soil-focused genome repositories and regionally curated databases, are expanding taxonomic representation and improving annotation accuracy (Bissett et al., 2016). In parallel, long-read sequencing, probe-based enrichment, and genome-resolved metagenomics are beginning to recover previously hidden bacterial clades from tropical soils (Tan et al., 2019). The persistence of a large unclassified fraction in the *H. arboreum* dataset nonetheless suggests that tropical coastal zones harbor substantial unexplored microbial diversity. Future work combining targeted cultivation, metagenome-assembled genome recovery, and database updating will be necessary to resolve these lineages and better link them to ecosystem function (Martí et al., 2025).

### 4.3 Shore-to-inland gradient and differential microbial responses

Rather than reiterating the richness pattern, our results suggest that the shore-to-inland transition acts as an environmental filter in *H. arboreum* habitats: reduced salinity stress and improved soil conditions inland likely expand available niches and strengthen plant–soil inputs, thereby promoting a more diverse rhizosphere microbiome. Nearshore zones typically experience strong environmental filtering through elevated salinity, coarse or unstable substrates, low organic matter, and limited water retention, all of which suppress microbial establishment and diversity. As distance from the sea increases, salinity stress declines, soils gradually accumulate organic matter, moisture regimes stabilize, and vegetation cover becomes denser, creating more favorable conditions for microbial proliferation and niche diversification. Comparable increases in bacterial and fungal alpha diversity from shoreline to inland areas have been observed in Mediterranean and Chinese coastal dunes, where reduced salt exposure and improved soil development coincide with richer microbial assemblages (Wasserstrom et al., 2017). Similar patterns occur in newly formed wetlands and coastal deltas, in which bacterial diversity rises progressively from saline margins toward freshwater or vegetated soils as nutrient availability, organic matter, and pH buffering improve (Li et al., 2019). Vegetation change reinforces this gradient by increasing litter and root-derived carbon inputs and by generating heterogeneous rhizosphere environments that support metabolically diverse microorganisms (Liu et al., 2008). The tight coupling between plant community complexity and microbial richness has been repeatedly documented across salinity and elevation gradients, where rhizodeposition and litter accumulation enhance microbial biomass and catabolic breadth alongside increasing vegetation structure (Zhang et al., 2015). In coastal successions more generally, salinity and nutrient gradients act as dominant drivers of microbial community composition, shaping both bacterial and fungal richness and promoting niche differentiation (Li et al., 2021). These studies indicate that the *H. arboreum* coastal–inland transect follows a well-established biogeographic pattern: as marine stress weakens inland and plant–soil interactions intensify, microbial richness and functional diversity increase accordingly.

The more pronounced spatial response of fungi likely reflects their stronger dependence on plant-derived carbon inputs and narrower tolerance to salinity and microhabitat disturbance, leading to greater community turnover along the shore-to-inland transition than is typically observed for bacteria. Fungal communities often display higher sensitivity to environmental gradients such as salinity, organic matter availability, and vegetation turnover, partly because many taxa depend strongly on plant-derived carbon and show lower physiological tolerance to osmotic stress (Li J. et al., 2022). In coastal wetlands, fungal richness and beta diversity typically rise sharply with distance from the sea as halophytic vegetation gives way to more diverse inland flora, increasing root exudation and organic carbon inputs (Dini-Andreote et al., 2016a). Bacterial communities, in contrast, generally show greater resilience to salinity and moisture fluctuations owing to higher physiological plasticity and broader niche breadth, leading to comparatively stable richness across coastal gradients (Chen et al., 2022). Even so, bacterial alpha diversity and functional potential still respond to inland shifts in pH, nitrate availability, and organic carbon accumulation, producing parallel but less pronounced spatial structuring (Song et al., 2024). Similar contrasts emerge in elevation and aridity studies, where fungi show higher turnover and narrower environmental tolerances than bacteria, indicating a stronger dependence on biotic drivers such as plant community composition (Chalmandrier et al., 2019) and vegetation biomass (Lange et al., 2014). These lines of evidence support the interpretation that fungal communities act as sensitive indicators of ecosystem transition, whereas bacterial communities reflect broader environmental adaptability, with both responding predictably to decreasing marine influence and increasing soil and vegetation development across the *H. arboreum* gradient.

### 4.4 Anthropogenic disturbance effects and indicator taxa

The distinctive fungal assemblages detected at human-impacted sites, particularly Wuzhizhou Island (a tourism-intensive location) and Danzhou (adjacent to a coastal highway), are consistent with broader evidence that anthropogenic disturbance is a major driver of microbial divergence in both coastal and terrestrial ecosystems. Human activities such as tourism, land-use change, and pollution commonly alter soil structure, salinity regimes, and nutrient availability, thereby creating novel microhabitats that favor opportunistic, disturbance-tolerant, or stress-adapted taxa (Chen et al., 2020). Such shifts often reduce overall fungal richness while increasing the relative dominance of a subset of taxa that can exploit fluctuating resources or withstand repeated physical disruption, producing more differentiated community structures among disturbed sites (Guajardo-Leiva et al., 2023). Because fungi depend strongly on vegetation-derived carbon and stable microclimatic conditions, their communities frequently show higher site-specificity under disturbance than bacteria. This pattern has been reported in human-modified island and coastal systems, including studies from the Galápagos and coastal vineyards, where fungal assemblages displayed stronger spatial distinctiveness and clearer disturbance signatures than bacterial communities (Liu et al., 2023; Schoenborn et al., 2023). In tourist and roadside environments, repeated trampling, localized nutrient deposition, and the introduction of non-native microbes can further intensify fungal compositional uniqueness (Lavoie et al., 2018). Together, these lines of evidence support the interpretation that in *H. arboreum* habitats, high-intensity human activity acts as an ecological filter that selects for distinct fungal communities, thereby increasing spatial differentiation relative to less disturbed sites.

The reduced abundance or loss of *Trichosporon* in seaward soils at Wuzhizhou Island (W), alongside increased representation of opportunistic or pathogenic guilds, further suggests a disturbance-mediated restructuring of fungal communities. In the *H. arboreum* coastal system, fungi appeared particularly sensitive to anthropogenic effects at tourism-influenced or shoreline-modified sites, where richness declined and community distinctiveness increased (Zhang et al., 2025). *Trichosporon* species are commonly reported as saprophytic yeasts in relatively undisturbed soils, but they can be sensitive to changes in substrate stability, nutrient inputs, and salinity, conditions that are frequently altered by trampling and recreational shoreline use. Comparable responses have been observed in other disturbed landscapes, where disturbance-sensitive genera, including *Trichoderma* and *Trichosporon*, decline under intensive land use while opportunistic taxa such as *Fusarium* and *Aspergillus* become more prevalent (Maina et al., 2016). Urban soil metabarcoding studies similarly show that human disturbance can elevate allergenic and pathogenic fungi (for example, *Alternaria*, *Bipolaris*, and *Fusarium*) while reducing saprotrophic taxa that contribute to soil health and decomposition processes (Marczylo et al., 2021). These findings align with a One Health perspective, in which disturbed soils may function as reservoirs of opportunistic pathogens with potential consequences for plant and human health (Yiallouris et al., 2024). In this context, *Trichosporon* may serve as a disturbance-sensitive indicator genus in *H. arboreum* systems, and its absence from heavily impacted habitats such as Wuzhizhou Island likely reflects a shift away from saprophytic balance toward communities increasingly structured by disturbance and pathogenic potential.

### 4.5 Environmental drivers of community structure (nutrients, pH, nitrate)

The significant associations between fungal community structure and soil physicochemical variables, especially available potassium (AK), total nitrogen (TN), and soil organic carbon (SOC), point to strong nutrient-based environmental filtering in *H. arboreum* rhizosphere fungi. In coastal soils, localized nutrient enrichment often promotes dominance by fast-growing saprotrophic or opportunistic genera such as *Fusarium* and *Aspergillus*, which are central to organic matter decomposition and nutrient turnover (Zhang et al., 2022). Similar redundancy analysis (RDA) studies identify TN, AK, and SOC as primary drivers of fungal differentiation, affecting both taxonomic composition and the distribution of functional guilds (Yu et al., 2023). *Fusarium* typically increases under higher TN and SOC, consistent with greater substrate availability for saprotrophic growth and for lineages with pathogenic potential (Huang et al., 2023). *Aspergillus* likewise responds positively to elevated AK and SOC, reflecting its involvement in potassium mineralization and its competitive success in nutrient-rich microsites (Bhattacharjee et al., 2021). These relationships suggest that organic matter accumulation and nutrient inputs not only increase fungal abundance but also shift community structure toward metabolically versatile taxa with high growth rates. Comparable patterns are reported in both managed and natural ecosystems, where increases in SOC and TN alter fungal community complexity and rebalance saprotrophic and pathogenic components (Wei et al., 2024). The coupled dynamics of AK, TN, and SOC appear to be major determinants of fungal composition in *H. arboreum* soils, with direct implications for decomposition intensity and coastal soil fertility.

Bacterial community composition in *H. arboreum* soils was most strongly related to soil pH and nitrate (NO_3_^−^), two widely recognized regulators of bacterial biogeography in coastal environments. Soil pH exerts pervasive control over bacterial diversity by shaping nutrient solubility, membrane stability, and enzyme function, and is repeatedly identified as a global predictor of bacterial alpha and beta diversity. Across wetlands, forests, and coastal gradients, neutral to mildly alkaline conditions commonly support higher bacterial richness and broader metabolic capability (Wang et al., 2023). In elevational and shoreline-to-inland transects, pH shifts often co-occur with nutrient redistribution, producing predictable changes in the relative dominance of phyla such as Proteobacteria, Acidobacteriota, and Actinobacteriota (Li M. et al., 2022). Nitrate availability further structures bacterial communities by functioning as both a nutrient source and an electron acceptor in nitrogen cycling. Taxa involved in nitrification and denitrification respond rapidly to nitrate enrichment, resulting in compositional and functional restructuring (Dini-Andreote et al., 2016b). Long-term studies show that NO_3_^−^ and pH can jointly account for a large share of bacterial turnover, emphasizing their combined influence on nitrogen transformation and ecosystem functioning (Yang et al., 2024). In the *H. arboreum* system, the dependence of bacterial composition on these variables suggests that modest shifts in soil chemistry, whether due to natural gradients or disturbance-driven nutrient inputs, may substantially change bacterial-mediated nutrient cycling and resilience.

### 4.6 Functional implications: nutrient cycling and disturbance-related pathogenic shifts

The co-dominance of *Fusarium* and *Stenotrophomonas* in the *H. arboreum* rhizosphere highlights complementary fungal–bacterial contributions to nutrient cycling in coastal soils. Although *Fusarium* is often discussed as a phytopathogen, it is also a major decomposer in natural systems, facilitating carbon and nitrogen turnover through enzymatic breakdown of complex residues. *Fusarium*, together with saprotrophic fungi such as *Aspergillus* and Stachybotrys, can accelerate litter decomposition and release labile carbon and nitrogen that support broader microbial activity (Zhang et al., 2023). In suppressive or nutrient-enriched soils, *Fusarium* may also shape microbial competition and, in some contexts, contribute indirectly to disease regulation by influencing community balance (Todorović et al., 2023). *Stenotrophomonas* species, in turn, are strongly linked to nitrogen metabolism, organic matter degradation, and plant growth promotion. Genomic evidence indicates that *Stenotrophomonas* contains genes for nitrogen fixation, phosphate solubilization, and polysaccharide degradation, traits that directly enhance nutrient cycling and plant nutrition (Zhao et al., 2024). Experimental work further suggests that *Stenotrophomonas maltophilia* can improve plant growth by strengthening nitrogen assimilation and reorganizing rhizosphere microbial networks (Sharma et al., 2025). Their concurrent dominance implies an efficient division of labor: *Fusarium* drives decomposition and carbon mineralization, while *Stenotrophomonas* supports nitrogen transformations and plant-associated nutrient acquisition. Such decomposer–nitrogen metabolizer partnerships are characteristic of residue-degrading consortia that stabilize carbon–nitrogen coupling under environmental stress (McClure et al., 2022). These patterns indicate that the *H. arboreum* rhizosphere sustains a functionally integrated microbial network that supports coastal soil fertility despite salinity and nutrient limitation.

The rhizosphere microbiome of *H. arboreum* appears functionally aligned with the demands of nutrient-poor, saline coastal habitats. Across sites, dominant bacterial and fungal taxa include decomposers, nitrogen-transformers, and organic-matter degraders, indicating a community structured to sustain carbon and nutrient turnover under chronic resource limitation. Recent metagenomic work from coastal Hainan supports this interpretation, showing enrichment of functionally versatile microbes in *H. arboreum* soils that can mediate nutrient transformation and carbon cycling despite oligotrophic and high-salinity conditions (Zhang et al., 2025). In the present dataset, bacterial genera such as *Stenotrophomonas* and *Bryobacter* were strongly associated with nitrogen availability, consistent with participation in nitrogen fixation and nitrification-linked processes. Fungal taxa including *Preussia* and *Metacordyceps* correlated positively with soil nutrient content, suggesting specialization in decomposition and carbon turnover. Similar functional backbones have been reported in arid and semi-arid rhizospheres, where nitrogen-fixing and carbon-degrading microbes underpin host persistence and productivity under resource stress (Li et al., 2025). The predominance of Proteobacteria and Ascomycota in *H. arboreum* soils is also consistent with global observations from coastal and desert ecosystems, in which metabolically flexible lineages dominate and confer resilience to fluctuating salinity and nutrient regimes (Ramond et al., 2022). Collectively, these patterns suggest that *H. arboreum* selectively assembles a functionally diverse microbiome that enhances nutrient cycling, stabilizes coastal substrates, and supports plant performance under environmental stress. Such functional complementarity between host and microbiome is consistent with broader evidence for long-term plant–microbe coadaptation in harsh environments (Ayangbenro et al., 2022).

Functional profiles also varied systematically with disturbance intensity. Sites subject to stronger human influence (W and D) exhibited higher predicted proportions of pathogenic functions, whereas less disturbed sites were dominated by saprophytic and nutrient-cycling guilds. This shift mirrors a widely documented global response in which disturbance reduces specialist taxa and favors opportunistic, stress-tolerant microbes, including potential pathogens (Seitz et al., 2021). Mechanistically, anthropogenic disturbance alters soil structure, nutrient dynamics, and microbial interactions in ways that favor pathogens. Soil compaction and vegetation loss disrupt aggregate stability and hyphal networks, increasing aeration variability and resource heterogeneity, which promotes the establishment of disturbance-tolerant spore-forming fungi. Nutrient inputs from runoff or organic debris enrich labile carbon and nitrogen pools, enhancing the competitiveness of fast-growing copiotrophs while suppressing slow-growing decomposers (Yan et al., 2025). Disturbance also weakens microbial competition and reduces community evenness, allowing opportunistic taxa such as *Fusarium* and *Aspergillus* to dominate (Wang and Kuzyakov, 2024). In *H. arboreum* habitats, enrichment of these genera at disturbed sites implies increased pathogenic potential and rapid exploitation of labile carbon pools, while undisturbed soils retain taxa such as *Mortierella* and *Stenotrophomonas* that sustain decomposition and nitrogen cycling. These processes create feedback loops in which plant decline and soil imbalance further reinforce pathogenic dominance (Encinas-Valero et al., 2024). Overall, even moderate human pressure can reorganize both microbial composition and function through soil degradation, nutrient enrichment, and altered competition dynamics, reshaping coastal ecosystems toward pathogen-enriched states.

## 5 Conclusion

This study reveals that *Heliotropium arboreum* supports distinct bacterial and fungal rhizosphere microbiomes structured by coastal environmental gradients and human disturbance across seven sites in Hainan. Across all sites, bacterial communities were consistently more diverse and compositionally stable, whereas fungal communities exhibited stronger turnover, indicating greater sensitivity to local environmental filtering. Rather than reflecting distance alone, the seaward-to-inland transition corresponds to a shift from high-stress, low-resource substrates to soils with greater organic inputs and improved nutrient conditions, which is consistent with the observed increase in microbial diversity, particularly for fungi. Environmental analyses further showed contrasting drivers for the two microbial groups: fungal community structure was most strongly associated with available potassium, total nitrogen, and soil organic carbon, while bacterial communities were primarily linked to soil pH and nitrate. Functional predictions indicated complementary roles in decomposition and nitrogen cycling, and sites with stronger human disturbance showed higher predicted pathogenic potential. Overall, the rhizosphere microbiome of *H. arboreum* appears to be environmentally filtered and functionally adaptive, supporting plant persistence in nutrient-poor coastal habitats under both natural gradients and anthropogenic pressure.

## Data Availability

The original contributions presented in the study are publicly available. This data can be found here: https://doi.org./10.6084/m9.figshare.30761966.

## References

[ref1] Ahkami A. H. White R. A. III Handakumbura P. P. Jansson C. (2017). Rhizosphere engineering: enhancing sustainable plant ecosystem productivity. Rhizosphere 3, 233–243. doi: 10.1016/j.rhisph.2017.04.012

[ref2] Anthony W. E. Allison S. D. Broderick C. M. Chavez Rodriguez L. Clum A. Cross H. . (2024). From soil to sequence: filling the critical gap in genome-resolved metagenomics is essential to the future of soil microbial ecology. Environ. Microb. 19:56. doi: 10.1186/s40793-024-00599-w, 39095861 PMC11295382

[ref3] Ayangbenro A. S. Chukwuneme C. F. Ayilara M. S. Kutu F. R. Khantsi M. Adeleke B. S. . (2022). Harnessing the rhizosphere soil microbiome of organically amended soil for plant productivity. Agron 12:3179. doi: 10.3390/agronomy12123179

[ref4] Barra Caracciolo A. Terenzi V. (2021). Rhizosphere microbial communities and heavy metals. Microorganisms 9:1462. doi: 10.3390/microorganisms9071462, 34361898 PMC8307176

[ref5] Bhattacharjee A. Qafoku O. Richardson J. A. Anderson L. N. Schwarz K. Bramer L. M. . (2021). Fungal mineral weathering mechanisms revealed through direct molecular visualization. bioRxiv. 2021–10. doi: 10.1101/2021.10.01.462718

[ref6] Bissett A. Fitzgerald A. Meintjes T. Mele P. M. Reith F. Dennis P. G. . (2016). Introducing BASE: the biomes of Australian soil environments soil microbial diversity database. Gigascience 5:21. doi: 10.1186/s13742-016-0126-5 27195106 PMC4870752

[ref7] Brunel C. Pouteau R. Dawson W. Pester M. Ramirez K. S. van Kleunen M. (2020). Towards unraveling macroecological patterns in rhizosphere microbiomes. Trends Plant Sci. 25, 1017–1029. doi: 10.1016/j.tplants.2020.04.015, 32467065

[ref8] Callahan B. J. McMurdie P. J. Rosen M. J. Han A. W. Johnson A. J. A. Holmes S. P. (2016). DADA2: high-resolution sample inference from Illumina amplicon data. Methods 13, 581–583. doi: 10.1038/nmeth.3869, 27214047 PMC4927377

[ref9] Chalmandrier L. Pansu J. Zinger L. Boyer F. Coissac E. Génin A. . (2019). Environmental and biotic drivers of soil microbial β-diversity across spatial and phylogenetic scales. Ecography 42, 2144–2156. doi: 10.1111/ecog.04492

[ref10] Chan K. G. Chong T. M. (2014). Prevalence of unclassified bacteria in tropical coastal waters of Malaysia revealed by metagenomic approach. Genome Announc. 2, 10–1128. doi: 10.1128/genomea.00419-14, 24812226 PMC4014694

[ref11] Chen Y. Kuang J. Wang P. Shu W. Barberan A. (2020). Associations between human impacts and forest soil microbial communities. Elem. Sci. Anth. 8:1. doi: 10.1525/elementa.005

[ref12] Chen Q. L. Xiang Q. Sun A. Q. Hu H. W. (2022). Aridity differentially alters the stability of soil bacterial and fungal networks in coastal and inland areas of Australia. Environ. Microbiol. 24, 5574–5582. doi: 10.1111/1462-2920.16186, 36070190 PMC9825871

[ref13] da Cunha I. D. C. M. da Silva A. V. R. Boleta E. H. M. Pellegrinetti T. A. Zagatto L. F. G. Zagatto S. D. S. S. . (2024). The interplay between the inoculation of plant growth-promoting rhizobacteria and the rhizosphere microbiome and their impact on plant phenotype. Microbiol. Res. 283:127706. doi: 10.1016/j.micres.2024.127706, 38574431

[ref14] Dai H. Jia M. Xue J. Liu Z. Zhou D. Hou Z. . (2025). Complementary rhizosphere microbial strategies drive functional specialization in coastal halophyte succession: differential adaptation of *Suaeda glauc*a and *Phragmites communis* to saline-alkali stress. Microorganisms. 13:1399. doi: 10.3390/microorganisms13061399, 40572287 PMC12195563

[ref15] Deng W. Chen S. Chen S. Xing B. Chan Z. Zhang Y. . (2024). Impacts of eutrophication on microbial community structure in sediment, seawater, and phyllosphere of seagrass ecosystems. Front. Microbiol. 15:1449545. doi: 10.3389/fmicb.2024.1449545, 39206368 PMC11350616

[ref16] Dini-Andreote F. Brossi M. J. D. L. Van Elsas J. D. Salles J. F. (2016b). Reconstructing the genetic potential of the microbially-mediated nitrogen cycle in a salt marsh ecosystem. Front. Microbiol. 7:902. doi: 10.3389/fmicb.2016.00902 27379042 PMC4908922

[ref17] Dini-Andreote F. Pylro V. S. Baldrian P. Van Elsas J. D. Salles J. F. (2016a). Ecological succession reveals potential signatures of marine–terrestrial transition in salt marsh fungal communities. ISME J. 10, 1984–1997. doi: 10.1038/ismej.2015.254, 26824176 PMC5029165

[ref18] Edwin N. R. Fitzpatrick A. H. Brennan F. Abram F. O’Sullivan O. (2024). An in-depth evaluation of metagenomic classifiers for soil microbiomes. Environ. Microb. 19:19. doi: 10.1186/s40793-024-00561-w, 38549112 PMC10979606

[ref19] Encinas-Valero M. Esteban R. Hereş A. M. Vivas M. Solla A. Moreno G. . (2024). Alteration of the tree–soil microbial system triggers a feedback loop that boosts holm oak decline. Funct. Ecol. 38, 374–390. doi: 10.1111/1365-2435.14473

[ref20] Fang F. Z. Chen S. L. Gui H. Y. Li Z. J. Zhang X. F. (2023). Long-read sequencing analysis revealed the impact of forest conversion on soil fungal diversity in Limu Mountain, Hainan[J]. Microb. Ecol. 86, 872–886. doi: 10.1007/s00248-022-02129-y 36329282

[ref21] Guajardo-Leiva S. Mendez K. N. Meneses C. Díez B. Castro-Nallar E. (2023). A first insight into the microbial and viral communities of Comau fjord—a unique human-impacted ecosystem in Patagonia (42 S). Microorganisms 11:904. doi: 10.3390/microorganisms11040904, 37110327 PMC10143455

[ref22] Guibert L. M. Loviso C. L. Borglin S. Jansson J. K. Dionisi H. M. Lozada M. (2016). Diverse bacterial groups contribute to the alkane degradation potential of chronically polluted subantarctic coastal sediments. Microb. Ecol. 71, 100–112. doi: 10.1007/s00248-015-0698-0, 26547568

[ref23] Hacquard S. Wang E. Slater H. Martin F. (2022). Impact of global change on the plant microbiome. New Phytol. 234, 1907–1909. doi: 10.1111/nph.18187 35599439

[ref24] Hol W. G. de Boer W. Medina A. (2014). “Beneficial interactions in the rhizosphere” in Interactions in Soil: promoting plant growth (Dordrecht: Springer Netherlands). 59–80.

[ref25] Huang A. Wang Z. Yang D. Yang S. Bai W. Wu N. . (2023). Effects of tea oil camellia (Camellia oleifera Abel.) shell-based organic fertilizers on the physicochemical property and microbial community structure of the rhizosphere soil. Front. Microbiol. 14:1231978. doi: 10.3389/fmicb.2023.1231978, 37637109 PMC10448393

[ref26] Klein M. Stewart J. D. Porter S. S. Weedon J. T. Kiers E. T. (2022). Evolution of manipulative microbial behaviors in the rhizosphere. Evol. Appl. 15, 1521–1536. doi: 10.1111/eva.13333, 36330300 PMC9624083

[ref27] Kõljalg U. Larsson K. H. Abarenkov K. Nilsson R. H. Alexander I. J. Eberhardt U. . (2005). UNITE: a database providing web-based methods for the molecular identification of ectomycorrhizal fungi. New Phytol. 166, 1063–1068. doi: 10.1111/j.1469-8137.2005.01376.x 15869663

[ref28] Kõljalg U. Nilsson R. H. Abarenkov K. Tedersoo L. Taylor A. F. Bahram M. . (2013). Towards a unified paradigm for sequence-based identification of Fungi. Mol. Ecol. 22, 5271–5277. doi: 10.1111/mec.12481 24112409

[ref29] Lange M. Habekost M. Eisenhauer N. Roscher C. Bessler H. Engels C. . (2014). Biotic and abiotic properties mediating plant diversity effects on soil microbial communities in an experimental grassland. PLoS One 9:e96182. doi: 10.1371/journal.pone.0096182, 24816860 PMC4015938

[ref30] Lavoie K. H. Winter A. S. Northup D. E. (2018). Impacts of indirect and direct visitation on microbial communities from lava caves in lava beds National Monument, USA. In Proceedings of the 18th International Symposium on Vulcanospeleology, Lava Beds National Monument, California, United States. International Union of Speleology, Volcanic Caves Commission.

[ref31] Li J. Cui L. Delgado-Baquerizo M. Wang J. Zhu Y. Wang R. . (2022). Fungi drive soil multifunctionality in the coastal salt marsh ecosystem. Sci. Total Environ. 818:151673. doi: 10.1016/j.scitotenv.2021.151673 34793796

[ref32] Li M. Dai G. Mu L. (2022). Composition and diversity of soil bacterial communities under identical vegetation along an elevational gradient in Changbai Mountains China. Front. Microbiol. 13:1065412. doi: 10.3389/fmicb.2022.1065412, 36532438 PMC9751831

[ref33] Li R. Jiao H. Sun B. Song M. Yan G. Bai Z. . (2024). Understanding salinity-driven modulation of microbial interactions: rhizosphere versus edaphic microbiome dynamics. Microorganisms 12:683. doi: 10.3390/microorganisms12040683 38674627 PMC11052110

[ref34] Li Y. Kang E. Song B. Wang J. Zhang X. Wang J. . (2021). Soil salinity and nutrients availability drive patterns in bacterial community and diversity along succession gradient in the Yellow River Delta. Estuar. Coast. Shelf Sci. 262:107621. doi: 10.1016/j.ecss.2021.107621

[ref35] Li C. Liu R. Tang L. Jiang L. (2025). Heavy-textured rhizosphere soils enhance microbial nitrogen fixation in a desert shrub ecosystem. Ecol. Evol. 15:e71210. doi: 10.1002/ece3.71210, 40212917 PMC11981880

[ref36] Li W. Lv X. Ruan J. Yu M. Song Y. B. Yu J. . (2019). Variations in soil bacterial composition and diversity in newly formed coastal wetlands. Front. Microbiol. 9:3256. doi: 10.3389/fmicb.2018.03256 30687257 PMC6333922

[ref37] Liu Y. Guo Z. Zhang P. Du J. Gao P. Zhang Z. (2022). Diversity and structure of vegetation rhizosphere bacterial community in various habitats of Liaohekou coastal wetlands. Sustainability 14:16396. doi: 10.3390/su142416396

[ref38] Liu M. Han X. Tong J. Zhu H. Bai X. (2020). Mutual environmental drivers of the community composition, functional attributes and co-occurrence patterns of bacterioplankton in the composite aquatic ecosystem of Taihu watershed in China. FEMS Microbiol. Ecol. 96:fiaa137. doi: 10.1093/femsec/fiaa137 32639543

[ref39] Liu M. Huang H. Bao S. Tong Y. (2019). Microbial community structure of soils in Bamenwan mangrove wetland. Sci. Rep. 9:8406. doi: 10.1038/s41598-019-44788-x, 31182804 PMC6557889

[ref40] Liu Z. Liu G. Fu B. Zheng X. (2008). Relationship between plant species diversity and soil microbial functional diversity along a longitudinal gradient in temperate grasslands of Hulunbeir, Inner Mongolia China. Ecol. Res. 23, 511–518. doi: 10.1007/s11284-007-0405-9

[ref41] Liu S. Xiong C. Lin L. Keyhani N. O. Zhu M. Zhao Z. . (2023). Assessing the structure and diversity of fungal community in plant soil under different climatic and vegetation conditions. Front. Microbiol. 14:1288066. doi: 10.3389/fmicb.2023.1288066, 38094633 PMC10716293

[ref42] López-Lozano N. E. Echeverría Molinar A. Ortiz Durán E. A. Hernández Rosales M. Souza V. (2020). Bacterial diversity and interaction networks of *Agave lechuguilla* rhizosphere differ significantly from bulk soil in the oligotrophic basin of *Cuatro Cienegas*. Front. Plant Sci. 11:1028. doi: 10.3389/fpls.2020.01028 32765547 PMC7378863

[ref43] Maina P. K. Wachira P. M. Okoth S. A. Kimenju J. W. Mwangi J. M. (2016). Co-occurrence and diversity of soil Trichoderma and *fusarium* species from different land use intensities in Machakos county Kenya. Arch. Current Res. Inter. 4, 1–13. doi: 10.9734/ACRI/2016/24894

[ref44] Marczylo E. L. Macchiarulo S. Gant T. W. (2021). Metabarcoding of soil fungi from different urban greenspaces around Bournemouth in the UK. EcoHealth 18, 315–330. doi: 10.1007/s10393-021-01523-1, 34089413 PMC8626400

[ref45] Martí J. M. Kok C. R. Thissen J. B. Mulakken N. J. Avila-Herrera A. Jaing C. J. . (2025). Addressing the dynamic nature of reference data: a new nucleotide database for robust metagenomic classification. mSystems 10, e01239–e01224. doi: 10.1128/msystems.01239-24 40111052 PMC12013259

[ref46] McClure R. Farris Y. Danczak R. Nelson W. Song H. S. Kessell A. . (2022). Interaction networks are driven by community-responsive phenotypes in a chitin-degrading consortium of soil microbes. Msystems 7, e0037222–e0037222. doi: 10.1128/msystems.00372-22, 36154140 PMC9599572

[ref47] Mommer L. Hinsinger P. Prigent-Combaret C. Visser E. J. (2016). Advances in the rhizosphere: stretching the interface of life. Plant Soil 407, 1–8. doi: 10.1007/s11104-016-3040-9

[ref48] Nguyen N. H. Song Z. Bates S. T. Branco S. Tedersoo L. Menke J. . (2016). FUNGuild: an open annotation tool for parsing fungal community datasets by ecological guild. Fungal Ecol. 20, 241–248. doi: 10.1016/j.funeco.2015.06.006

[ref49] Portalanza D. Acosta-Mejillones A. Alcívar J. Colorado T. Guaita J. Montero L. . (2025). Fungal community dynamics in *Cyperus rotundus*: implications for *Rhizophora mangle* in a mangrove ecosystem. Int. J. Plant Biol. 16:23. doi: 10.3390/ijpb16010023

[ref50] Qiu L. Kong W. Zhu H. Zhang Q. Banerjee S. Ishii S. . (2021). Halophytes increase rhizosphere microbial diversity and network complexity in inland saline ecosystem. Sci. Total Environ. 831:154944. doi: 10.1016/j.scitotenv.2022.154944 35367547

[ref51] Quast C. Pruesse E. Yilmaz P. Gerken J. Schweer T. Yarza P. . (2012). The SILVA ribosomal RNA gene database project: improved data processing and web-based tools. Nucleic Acids Res. 41, D590–D596. doi: 10.1093/nar/gks1219, 23193283 PMC3531112

[ref52] Ramond J. B. Jordaan K. Díez B. Heinzelmann S. M. Cowan D. A. (2022). Microbial biogeochemical cycling of nitrogen in arid ecosystems. Microbiol. Mol. Biol. Rev. 86, e0010921–e0010921. doi: 10.1128/mmbr.00109-21, 35389249 PMC9199420

[ref53] Rath K. M. Fierer N. Murphy D. V. Rousk J. (2019). Linking bacterial community composition to soil salinity along environmental gradients. ISME J. 13, 836–846. doi: 10.1038/s41396-018-0313-8, 30446737 PMC6461869

[ref54] Schiro G. Chen Y. Blankinship J. C. Barberán A. (2022). Ride the dust: linking dust dispersal and spatial distribution of microorganisms across an arid landscape. Environ. Microbiol. 24, 4094–4107. doi: 10.1111/1462-2920.15998, 35384241

[ref55] Schoenborn A. A. Yannarell S. M. MacVicar C. T. Barriga-Medina N. N. Bonham K. S. Leon-Reyes A. . (2023). Microclimate is a strong predictor of the native and invasive plant-associated soil microbiome on San Cristóbal Island, Galápagos archipelago. &lt;i&gt;environmental microbiology&lt;/i&gt. Environ. Microbiol. 25, 1377–1392. doi: 10.1111/1462-2920.16361 36883264

[ref56] Seitz T. J. Schütte U. M. Drown D. M. (2021). Soil disturbance affects plant productivity via soil microbial community shifts. Front. Microbiol. 12:619711. doi: 10.3389/fmicb.2021.619711, 33597939 PMC7882522

[ref57] Sharma P. Pandey R. Chauhan N. S. (2025). *Stenotrophomonas maltophilia* promotes wheat growth by enhancing nutrient assimilation and rhizosphere microbiota modulation. Front. Bioeng. Biotechnol. 13:1563670. doi: 10.3389/fbioe.2025.1563670, 40313642 PMC12043639

[ref58] Song B. Wang T. Wan C. Cai Y. Mao L. Ge Z. . (2024). Diversity patterns and drivers of soil bacterial and fungal communities in a muddy coastal wetland of China. J. Fungi 10:770. doi: 10.3390/jof10110770, 39590689 PMC11595316

[ref59] Tan S. Yung P. Hutchinson P. Xie C. Teo G. Ismail M. . (2019). Primer-free FISH probes from metagenomics/metatranscriptomics data permit the study of uncharacterised taxa in complex microbial communities. NPJ Biofilms Microb. 5:17. doi: 10.1038/s41522-019-0090-9, 31263569 PMC6592924

[ref60] Thomson T. Fusi M. Bennett-Smith M. F. Prinz N. Aylagas E. Carvalho S. . (2022). Contrasting effects of local environmental and biogeographic factors on the composition and structure of bacterial communities in arid monospecific mangrove soils. Microbiol. Spectr. 10, e00903–e00921. doi: 10.1128/spectrum.00903-21, 34985338 PMC8729789

[ref61] Todorović I. Moënne-Loccoz Y. Raičević V. Jovičić-Petrović J. Muller D. (2023). Microbial diversity in soils suppressive to *fusarium* diseases. Front. Plant Sci. 14:1228749. doi: 10.3389/fpls.2023.1228749, 38111879 PMC10726057

[ref62] Tugbaeva A. S. Ermoshin A. A. Shiryaev G. I. Kiseleva I. S. (2025). Microbiome of the soil and rhizosphere of the halophyte *Spergularia marina* (L.) Griseb in the saline sites of Lake Kurgi, the south Urals: metagenomic analysis. Microbiol. Res. 16:64. doi: 10.3390/microbiolres16030064

[ref63] Turner B. L. Laliberté E. (2015). Soil development and nutrient availability along a 2 million-year coastal dune chronosequence under species-rich Mediterranean shrubland in southwestern Australia. Ecosystems 18, 287–309. doi: 10.1007/s10021-014-9830-0

[ref64] Wang C. Kuzyakov Y. (2024). Mechanisms and implications of bacterial–fungal competition for soil resources. ISME J. 18:wrae073. doi: 10.1093/ismejo/wrae073, 38691428 PMC11104273

[ref65] Wang M. Pu W. Wang S. Zeng X. Sui X. Wang X. (2023). pH-related changes in soil bacterial communities in the Sanjiang plain, Northeast China. Microorganisms 11:2950. doi: 10.3390/microorganisms11122950, 38138094 PMC10745975

[ref66] Ward T. Larson J. Meulemans J. Hillmann B. Lynch J. Sidiropoulos D. . (2017). BugBase predicts organism-level microbiome phenotypes. bioRxiv.:133462. doi: 10.1101/133462

[ref67] Wasserstrom H. Kublik S. Wasserstrom R. Schulz S. Schloter M. Steinberger Y. (2017). Bacterial community composition in costal dunes of the Mediterranean along a gradient from the sea shore to the inland. Sci. Rep. 7:40266. doi: 10.1038/srep40266, 28074923 PMC5225477

[ref68] Wei T. J. Li G. Cui Y. R. Xie J. Liang Z. W. Guan F. C. . (2024). Response of alfalfa leaf traits and rhizosphere fungal communities to compost application in saline–sodic soil. Microorganisms 12:2287. doi: 10.3390/microorganisms12112287, 39597677 PMC11596975

[ref69] Wu T. Sabula M. Milner H. Strickland G. Liu G. (2023). Agricultural practice negatively affects soil bacterial diversity and nitrogen functional genes comparing to adjacent native forest soils. Appl. Soil Ecol. 186:104856. doi: 10.1016/j.apsoil.2023.104856

[ref70] Yan Y. Zhou X. Liu L. Cai Z. Penuelas J. Huang X. (2025). Soil nutrient enrichment induces trade-offs in bacterial life-history strategies promoting plant productivity. Adv. Sci. 12:e10066. doi: 10.1002/advs.202510066, 40966389 PMC12677649

[ref71] Yang Y. Li Y. Hao K. Zhao Y. Li M. Fan Y. (2024). Microbial community composition and co-occurrence network analysis of the rhizosphere soil of the main constructive tree species in Helan Mountain of Northwest China. Sci. Rep. 14:24557. doi: 10.1038/s41598-024-76195-2, 39427091 PMC11490567

[ref72] Yiallouris A. Pana Z. D. Marangos G. Tzyrka I. Karanasios S. Georgiou I. . (2024). Fungal diversity in the soil Mycobiome: implications for ONE health. One Health 18:100720. doi: 10.1016/j.onehlt.2024.100720, 38699438 PMC11064618

[ref73] Yu J. Li S. Sun X. Zhou W. He L. Zhao G. . (2023). The impact and determinants of mountainous topographical factors on soil microbial community characteristics. Microorganisms 11:2878. doi: 10.3390/microorganisms11122878, 38138022 PMC10746091

[ref74] Zhang Y. Cong J. Lu H. Li G. Xue Y. Deng Y. . (2015). Soil bacterial diversity patterns and drivers along an elevational gradient on Shennongjia mountain China. Microb. Biotechnol. 8, 739–746. doi: 10.1111/1751-7915.12288, 26032124 PMC4476828

[ref75] Zhang X. F. Fang F. Z. Sha L. H. Nizamani M. M. (2025). Ecological dynamics and microbial community composition of *Heliotropium arboreum* in the coastal ecosystems of Hainan province. Front. Microbiol. 16:1611262. doi: 10.3389/fmicb.2025.1611262 40678047 PMC12267182

[ref76] Zhang L. Li J. Wang Z. Zhang D. Liu H. Wang J. . (2023). Litter mixing promoted decomposition and altered microbial community in common bean root litter. BMC Microbiol. 23:148. doi: 10.1186/s12866-023-02871-4, 37217839 PMC10204263

[ref77] Zhang M. Liu Y. Wei Q. Gu X. Liu L. Gou J. (2022). Biochar application ameliorated the nutrient content and fungal community structure in different yellow soil depths in the karst area of Southwest China. Front. Plant Sci. 13:1020832. doi: 10.3389/fpls.2022.1020832, 36352867 PMC9638009

[ref78] Zhao Y. Ding W. J. Xu L. Sun J. Q. (2024). A comprehensive comparative genomic analysis revealed that plant growth promoting traits are ubiquitous in strains of Stenotrophomonas. Front. Microbiol. 15:1395477. doi: 10.3389/fmicb.2024.1395477, 38817968 PMC11138164

[ref79] Zhou X. Dong S. Li Y. Ding W. Shen H. Gai Y. . (2025). Increasing nitrogen deposition promotes recruitment of beneficial rhizosphere microbes to help grass species dominate Plant Community of Alpine Meadow on Qinghai-Tibetan plateau. Grass Forage Sci. 80:e12726. doi: 10.1111/gfs.12726

