# Supplementary material for: Coastal gradients and human disturbance shape bacterial and fungal rhizosphere microbiomes of Heliotropium arboreum in Hainan, China
Source: Front Microbiol. 2026 Feb 2;17:1774048. doi: 10.3389/fmicb.2026.1774048 (PMC12907427; doi:10.3389/fmicb.2026.1774048)
Supplement: Supplementary file 3 [file Table_3.DOCX]

**Table S3 Sequencing data of fungal samples**

| **SampleID** | **Raw reads** | **Clean reads** | **Effective reads** | **Q20(%)** | **Q30(%)** | **GC(%)** |
| --- | --- | --- | --- | --- | --- | --- |
| Aa1 | 85731 | 85640 | 84131 | 100 | 99.8 | 43.2 |
| Aa2 | 89037 | 88900 | 87388 | 100 | 99.8 | 42.6 |
| Aa3 | 84896 | 84795 | 83574 | 100 | 99.7 | 42.2 |
| Ab1 | 87004 | 86842 | 83324 | 99.9 | 99.4 | 46.8 |
| Ab2 | 84285 | 84132 | 81299 | 99.9 | 99.5 | 47.8 |
| Ab3 | 85060 | 84868 | 80123 | 99.9 | 99.5 | 46.8 |
| Ac1 | 90767 | 90674 | 88595 | 99.9 | 99.6 | 48.5 |
| Ac2 | 91135 | 91078 | 87714 | 99.9 | 99.4 | 49.3 |
| Ac3 | 85907 | 85827 | 84070 | 99.9 | 99.5 | 48.6 |
| Ba1 | 88030 | 87929 | 85205 | 99.9 | 99.4 | 42.5 |
| Ba2 | 84356 | 84131 | 76244 | 99.9 | 99.4 | 45.7 |
| Ba3 | 86860 | 86785 | 82706 | 99.9 | 99.4 | 41.7 |
| Bb1 | 85037 | 84929 | 80753 | 99.9 | 99.5 | 47.9 |
| Bb2 | 91100 | 91010 | 87276 | 99.9 | 99.5 | 46.9 |
| Bb3 | 88387 | 88303 | 84120 | 99.9 | 99.5 | 47.4 |
| Bc1 | 85197 | 84284 | 78489 | 99.9 | 99.4 | 47.9 |
| Bc2 | 84176 | 84008 | 82032 | 99.9 | 99.7 | 45.8 |
| Bc3 | 90443 | 89937 | 84573 | 99.9 | 99.5 | 47.6 |
| Ca1 | 86999 | 86884 | 84926 | 99.9 | 99.5 | 45.6 |
| Ca2 | 74515 | 74448 | 73216 | 99.9 | 99.5 | 46.1 |
| Ca3 | 91537 | 91451 | 89217 | 99.9 | 99.5 | 44.5 |
| Cb1 | 79652 | 79596 | 77496 | 99.9 | 99.5 | 50.8 |
| Cb2 | 86128 | 86048 | 83364 | 99.9 | 99.5 | 51 |
| Cb3 | 88404 | 88334 | 85400 | 99.9 | 99.4 | 50.7 |
| Cc1 | 88585 | 88372 | 85284 | 99.7 | 98.5 | 47.2 |
| Cc2 | 91374 | 91271 | 88892 | 99.9 | 99.4 | 46.4 |
| Cc3 | 85351 | 85218 | 83074 | 99.9 | 99.3 | 46.1 |
| Da1 | 91579 | 91484 | 89478 | 99.9 | 99.4 | 46.9 |
| Da2 | 89057 | 88950 | 86491 | 99.8 | 99.1 | 45.5 |
| Da3 | 72508 | 72425 | 70576 | 99.8 | 99 | 47.3 |
| Db1 | 86222 | 86153 | 83789 | 99.9 | 99.5 | 50.1 |
| Db2 | 91072 | 90998 | 88571 | 99.9 | 99.6 | 49 |
| Db3 | 91210 | 91146 | 89341 | 99.9 | 99.5 | 50.1 |
| Dc1 | 92299 | 92230 | 90087 | 99.9 | 99.7 | 48.2 |
| Dc2 | 84517 | 84466 | 83379 | 100 | 99.8 | 48 |
| Dc3 | 88188 | 88111 | 86268 | 99.9 | 99.6 | 50.1 |
| La1 | 55968 | 55903 | 54780 | 99.9 | 99.5 | 45.2 |
| La2 | 84642 | 84407 | 82270 | 99.9 | 99.5 | 44.7 |
| La3 | 79643 | 79566 | 78087 | 99.9 | 99.5 | 43.3 |
| Lb1 | 76309 | 76220 | 74232 | 99.9 | 99.6 | 46.9 |
| Lb2 | 90405 | 90323 | 86501 | 99.9 | 99.5 | 46 |
| Lb3 | 85609 | 85537 | 82347 | 99.9 | 99.5 | 46.1 |
| Lc1 | 71899 | 71838 | 70272 | 99.9 | 99.6 | 46.5 |
| Lc2 | 87034 | 86966 | 85711 | 100 | 99.8 | 51.8 |
| Lc3 | 86898 | 86833 | 83997 | 99.8 | 99.1 | 46.3 |
| Ma1 | 86769 | 86672 | 83965 | 99.9 | 99.2 | 43.3 |
| Ma2 | 86141 | 86051 | 82029 | 99.7 | 98.8 | 44.4 |
| Ma3 | 89595 | 89500 | 87028 | 99.9 | 99.3 | 44.5 |
| Mb1 | 87300 | 87232 | 84772 | 99.9 | 99.7 | 49 |
| Mb2 | 87685 | 87633 | 83930 | 99.9 | 99.6 | 50.2 |
| Mb3 | 88273 | 88206 | 85874 | 99.9 | 99.6 | 50.6 |
| Mc1 | 85358 | 85288 | 83249 | 99.9 | 99.6 | 49.7 |
| Mc2 | 85547 | 85486 | 84116 | 99.9 | 99.6 | 49.7 |
| Mc3 | 92065 | 91970 | 88808 | 99.9 | 99.5 | 48.6 |
| Wa1 | 91305 | 91202 | 89571 | 99.9 | 99.6 | 42.8 |
| Wa2 | 90618 | 90525 | 88917 | 99.9 | 99.7 | 42.5 |
| Wa3 | 89445 | 89326 | 87783 | 99.9 | 99.6 | 42.7 |
| Wb1 | 84446 | 84383 | 81983 | 99.9 | 99.2 | 56.3 |
| Wb2 | 91735 | 91624 | 88680 | 99.9 | 99.5 | 45.8 |
| Wb3 | 86884 | 86792 | 84136 | 99.6 | 98.2 | 43 |
| Wc1 | 85015 | 84892 | 82056 | 99.9 | 99.5 | 46.8 |
| Wc2 | 84741 | 84613 | 81391 | 99.9 | 99.5 | 44.7 |
| Wc3 | 90494 | 90409 | 88132 | 99.9 | 99.6 | 48.3 |
