# Supplementary material for: Coastal gradients and human disturbance shape bacterial and fungal rhizosphere microbiomes of Heliotropium arboreum in Hainan, China
Source: Front Microbiol. 2026 Feb 2;17:1774048. doi: 10.3389/fmicb.2026.1774048 (PMC12907427; doi:10.3389/fmicb.2026.1774048)
Supplement: Supplementary file 4 [file Table_4.DOCX]

**Table S4 Sequencing data of bacterial samples**

| **SampleID** | **Raw reads** | **Clean reads** | **Effective reads** | **Q20(%)** | **Q30(%)** | **GC(%)** |
| --- | --- | --- | --- | --- | --- | --- |
| Aa1 | 87492 | 87423 | 86376 | 99.9 | 99.5 | 54.3 |
| Aa2 | 88014 | 87947 | 86959 | 99.9 | 99.6 | 54.4 |
| Aa3 | 87231 | 87147 | 85961 | 99.9 | 99.5 | 54.3 |
| Ab1 | 85905 | 85823 | 84795 | 99.9 | 99.6 | 56.3 |
| Ab2 | 65819 | 65747 | 64921 | 99.9 | 99.5 | 56.5 |
| Ab3 | 54153 | 54110 | 53520 | 99.9 | 99.6 | 56.6 |
| Ac1 | 78503 | 78414 | 77465 | 99.9 | 99.6 | 56.9 |
| Ac2 | 85944 | 85854 | 84812 | 99.9 | 99.6 | 56.8 |
| Ac3 | 50987 | 50929 | 50355 | 99.9 | 99.5 | 56.8 |
| Ba1 | 88945 | 88855 | 87719 | 99.9 | 99.6 | 55.3 |
| Ba2 | 87140 | 87053 | 85939 | 99.9 | 99.5 | 55.5 |
| Ba3 | 87958 | 87873 | 86868 | 99.9 | 99.6 | 55.2 |
| Bb1 | 88075 | 87997 | 86855 | 99.9 | 99.5 | 56.1 |
| Bb2 | 84501 | 84417 | 83415 | 99.9 | 99.5 | 56.2 |
| Bb3 | 85607 | 85512 | 84360 | 99.9 | 99.5 | 56.1 |
| Bc1 | 87409 | 87304 | 86202 | 99.9 | 99.5 | 56.6 |
| Bc2 | 84981 | 84883 | 83752 | 99.9 | 99.5 | 56.1 |
| Bc3 | 84778 | 84687 | 83475 | 99.9 | 99.5 | 56.4 |
| Ca1 | 91083 | 90991 | 89765 | 99.9 | 99.5 | 54.3 |
| Ca2 | 86668 | 86564 | 85473 | 99.9 | 99.5 | 54.2 |
| Ca3 | 90164 | 90074 | 88915 | 99.9 | 99.5 | 53.9 |
| Cb1 | 89590 | 89505 | 88360 | 99.9 | 99.5 | 55.7 |
| Cb2 | 88173 | 88090 | 86917 | 99.9 | 99.5 | 56.2 |
| Cb3 | 90445 | 90352 | 89096 | 99.9 | 99.5 | 56 |
| Cc1 | 88580 | 88479 | 87274 | 99.9 | 99.6 | 56.3 |
| Cc2 | 92333 | 92216 | 90944 | 99.9 | 99.5 | 56.4 |
| Cc3 | 92269 | 92161 | 90960 | 99.9 | 99.5 | 56.4 |
| Da1 | 87041 | 86934 | 85699 | 99.9 | 99.5 | 53.7 |
| Da2 | 90662 | 90578 | 89228 | 99.9 | 99.5 | 54.1 |
| Da3 | 85014 | 84935 | 83702 | 99.9 | 99.5 | 54.7 |
| Db1 | 85435 | 85352 | 84320 | 99.9 | 99.5 | 57 |
| Db2 | 89249 | 89154 | 87735 | 99.9 | 99.5 | 56.8 |
| Db3 | 87950 | 87857 | 86836 | 99.9 | 99.5 | 56.9 |
| Dc1 | 91851 | 91760 | 90581 | 99.9 | 99.5 | 54.9 |
| Dc2 | 89800 | 89718 | 88717 | 99.9 | 99.5 | 54.5 |
| Dc3 | 86095 | 85971 | 84770 | 99.9 | 99.5 | 54.8 |
| La1 | 87008 | 86911 | 85794 | 99.9 | 99.5 | 54.8 |
| La2 | 90974 | 90861 | 89631 | 99.9 | 99.5 | 54.8 |
| La3 | 59920 | 59854 | 59057 | 99.9 | 99.5 | 54.7 |
| Lb1 | 86735 | 86649 | 85545 | 99.9 | 99.6 | 56 |
| Lb2 | 88253 | 88159 | 87049 | 99.9 | 99.6 | 55.9 |
| Lb3 | 88393 | 88285 | 87099 | 99.9 | 99.5 | 56.2 |
| Lc1 | 86975 | 86892 | 85788 | 99.9 | 99.5 | 55.4 |
| Lc2 | 88223 | 88119 | 86890 | 99.9 | 99.5 | 55.8 |
| Lc3 | 83739 | 83654 | 82712 | 99.9 | 99.5 | 55.2 |
| Ma1 | 89016 | 88918 | 87728 | 99.9 | 99.5 | 54.7 |
| Ma2 | 90166 | 90063 | 88725 | 99.9 | 99.5 | 54.4 |
| Ma3 | 88905 | 88807 | 87754 | 99.9 | 99.6 | 54.4 |
| Mb1 | 85536 | 85453 | 84236 | 99.9 | 99.5 | 55.3 |
| Mb2 | 91109 | 91015 | 89790 | 99.9 | 99.5 | 55.6 |
| Mb3 | 86293 | 86211 | 84941 | 99.9 | 99.5 | 55.7 |
| Mc1 | 86459 | 86351 | 85127 | 99.9 | 99.5 | 56.8 |
| Mc2 | 92114 | 91972 | 90603 | 99.9 | 99.5 | 56.7 |
| Mc3 | 89826 | 89728 | 88498 | 99.9 | 99.5 | 56.9 |
| Wa1 | 92338 | 92242 | 91038 | 99.9 | 99.5 | 54.2 |
| Wa2 | 88544 | 88436 | 87103 | 99.9 | 99.5 | 54.2 |
| Wa3 | 85389 | 85290 | 83982 | 99.9 | 99.5 | 54.3 |
| Wb1 | 91007 | 90913 | 89763 | 99.8 | 99.1 | 54.9 |
| Wb2 | 56129 | 56076 | 55075 | 99.9 | 99.4 | 56 |
| Wb3 | 91164 | 91064 | 89881 | 99.9 | 99.5 | 55.3 |
| Wc1 | 84169 | 84050 | 82942 | 99.9 | 99.5 | 56.5 |
| Wc2 | 86119 | 86009 | 84732 | 99.9 | 99.5 | 56.5 |
| Wc3 | 84938 | 84825 | 83501 | 99.9 | 99.5 | 56.6 |
