# Supplementary material for: Coastal gradients and human disturbance shape bacterial and fungal rhizosphere microbiomes of Heliotropium arboreum in Hainan, China
Source: Front Microbiol. 2026 Feb 2;17:1774048. doi: 10.3389/fmicb.2026.1774048 (PMC12907427; doi:10.3389/fmicb.2026.1774048)
Supplement: Supplementary file 5 [file Table_5.DOCX]

**Table S5 Statistics of OTU and alpha diversity index of fungi**

| **Sample** | **OTUs** | **Richness** | **Chao1** | **Shannon** | **ACE** |
| --- | --- | --- | --- | --- | --- |
| Aa1 | 236 | 59 | 61.5 | 1.87 | 90.42 |
| Aa2 | 268 | 56 | 59.8 | 2.38 | 87.67 |
| Aa3 | 271 | 52 | 54.3 | 3.37 | 68.76 |
| Ab1 | 391 | 78 | 78.5 | 1.61 | 82.94 |
| Ab2 | 418 | 86 | 86.9 | 1.96 | 99.89 |
| Ab3 | 403 | 81 | 85.1 | 1.47 | 102.41 |
| Ac1 | 392 | 85 | 85.5 | 2.75 | 91.12 |
| Ac2 | 383 | 82 | 87.1 | 2.24 | 98.28 |
| Ac3 | 394 | 85 | 88.9 | 2.76 | 97.54 |
| Ba1 | 348 | 83 | 86.1 | 1.46 | 120.22 |
| Ba2 | 414 | 85 | 87 | 2.43 | 100.02 |
| Ba3 | 424 | 89 | 90 | 2.1 | 113.29 |
| Bb1 | 377 | 80 | 81.5 | 2.95 | 95.27 |
| Bb2 | 413 | 93 | 97 | 3.23 | 122.00 |
| Bb3 | 400 | 88 | 89.3 | 3.31 | 102.22 |
| Bc1 | 352 | 80 | 86.5 | 3.32 | 96.34 |
| Bc2 | 402 | 86 | 88.7 | 2.6 | 98.60 |
| Bc3 | 372 | 77 | 77.7 | 3.1 | 84.28 |
| Ca1 | 367 | 73 | 75.3 | 1.42 | 100.90 |
| Ca2 | 386 | 74 | 78.5 | 2.23 | 101.08 |
| Ca3 | 328 | 66 | 67.5 | 2.27 | 86.76 |
| Cb1 | 472 | 98 | 99.6 | 2.63 | 120.37 |
| Cb2 | 409 | 96 | 97.1 | 2.26 | 126.19 |
| Cb3 | 432 | 80 | 84.5 | 2.23 | 105.12 |
| Cc1 | 282 | 81 | 81.5 | 2.2 | 82.41 |
| Cc2 | 304 | 78 | 78.9 | 2.35 | 101.26 |
| Cc3 | 320 | 79 | 83.5 | 2.15 | 116.27 |
| Da1 | 399 | 89 | 93.2 | 2.31 | 132.48 |
| Da2 | 286 | 67 | 68.5 | 2.34 | 89.40 |
| Da3 | 242 | 55 | 55.7 | 2.87 | 68.60 |
| Db1 | 391 | 100 | 111.5 | 2.77 | 139.64 |
| Db2 | 392 | 91 | 93.7 | 2.4 | 104.76 |
| Db3 | 388 | 101 | 105.9 | 2.76 | 147.27 |
| Dc1 | 457 | 117 | 128.3 | 3.67 | 142.63 |
| Dc2 | 427 | 112 | 113.7 | 1.72 | 138.82 |
| Dc3 | 381 | 99 | 100.1 | 3.19 | 111.29 |
| La1 | 266 | 62 | 65.4 | 2.24 | 109.39 |
| La2 | 371 | 84 | 86.1 | 2.43 | 116.19 |
| La3 | 314 | 74 | 74.7 | 1.73 | 96.61 |
| Lb1 | 377 | 93 | 97.2 | 2.57 | 139.96 |
| Lb2 | 406 | 100 | 102.2 | 2.65 | 118.60 |
| Lb3 | 401 | 91 | 93.3 | 2.11 | 109.62 |
| Lc1 | 365 | 81 | 90.7 | 2.66 | 106.55 |
| Lc2 | 448 | 92 | 93.1 | 1.25 | 108.41 |
| Lc3 | 285 | 79 | 79.2 | 2.75 | 79.62 |
| Ma1 | 382 | 83 | 87.5 | 3.17 | 105.11 |
| Ma2 | 233 | 70 | 70.8 | 2.8 | 71.97 |
| Ma3 | 283 | 66 | 67.8 | 2.41 | 88.17 |
| Mb1 | 369 | 84 | 85 | 2.37 | 96.87 |
| Mb2 | 352 | 85 | 86.1 | 2.96 | 95.00 |
| Mb3 | 367 | 87 | 88 | 2.43 | 101.57 |
| Mc1 | 487 | 115 | 117.7 | 2.81 | 141.49 |
| Mc2 | 510 | 107 | 108.3 | 2.35 | 118.54 |
| Mc3 | 498 | 110 | 142 | 3.31 | 126.54 |
| Wa1 | 319 | 65 | 65.8 | 0.902 | 80.84 |
| Wa2 | 377 | 77 | 80.4 | 0.467 | 103.42 |
| Wa3 | 365 | 84 | 85.5 | 0.57 | 112.22 |
| Wb1 | 329 | 81 | 81.9 | 1.25 | 88.88 |
| Wb2 | 395 | 88 | 92.5 | 1.72 | 120.59 |
| Wb3 | 370 | 107 | 107 | 1.04 | 107.30 |
| Wc1 | 454 | 102 | 102.4 | 2.27 | 112.99 |
| Wc2 | 490 | 101 | 102 | 2.07 | 116.21 |
| Wc3 | 458 | 92 | 97 | 2.63 | 112.99 |
