# Supplementary material for: Coastal gradients and human disturbance shape bacterial and fungal rhizosphere microbiomes of Heliotropium arboreum in Hainan, China
Source: Front Microbiol. 2026 Feb 2;17:1774048. doi: 10.3389/fmicb.2026.1774048 (PMC12907427; doi:10.3389/fmicb.2026.1774048)
Supplement: Supplementary file 6 [file Table_6.DOCX]

**Table S6 Statistics of bacterial OTU and alpha diversity index**

| **Sample** | **OTUs** | **Richness** | **Chao1** | **Shannon_2** | **ACE** |
| --- | --- | --- | --- | --- | --- |
| Aa1 | 5265 | 288 | 289.5 | 3.31 | 347.86 |
| Aa2 | 5861 | 285 | 288.8 | 3.07 | 339.35 |
| Aa3 | 5266 | 271 | 272.5 | 3.21 | 314.20 |
| Ab1 | 4671 | 233 | 234.5 | 2.81 | 265.69 |
| Ab2 | 4502 | 242 | 243 | 2.69 | 273.54 |
| Ab3 | 4131 | 214 | 214.9 | 2.7 | 245.32 |
| Ac1 | 4786 | 260 | 261 | 2.41 | 294.42 |
| Ac2 | 4879 | 227 | 227.9 | 2.35 | 246.53 |
| Ac3 | 3992 | 218 | 219.2 | 2.62 | 240.93 |
| Ba1 | 3118 | 222 | 225.1 | 2.94 | 277.71 |
| Ba2 | 3015 | 200 | 201.3 | 2.88 | 224.70 |
| Ba3 | 3165 | 225 | 226.9 | 2.97 | 289.05 |
| Bb1 | 4806 | 277 | 279.5 | 2.57 | 342.97 |
| Bb2 | 5154 | 305 | 307.3 | 2.84 | 362.53 |
| Bb3 | 4981 | 285 | 286.4 | 2.73 | 328.08 |
| Bc1 | 4587 | 238 | 239.9 | 2.37 | 284.46 |
| Bc2 | 4699 | 283 | 284 | 2.81 | 320.20 |
| Bc3 | 4646 | 242 | 244.1 | 2.56 | 280.17 |
| Ca1 | 5679 | 299 | 299.8 | 2.84 | 341.96 |
| Ca2 | 5451 | 275 | 276.4 | 2.82 | 320.28 |
| Ca3 | 5735 | 311 | 311.9 | 3.13 | 355.14 |
| Cb1 | 4905 | 287 | 289.6 | 3 | 327.82 |
| Cb2 | 5042 | 279 | 279.9 | 2.87 | 309.72 |
| Cb3 | 5220 | 301 | 301.4 | 3.09 | 316.99 |
| Cc1 | 3814 | 206 | 207.3 | 2.46 | 239.36 |
| Cc2 | 4111 | 219 | 219.7 | 2.49 | 262.79 |
| Cc3 | 4051 | 220 | 221.3 | 2.56 | 259.77 |
| Da1 | 7021 | 363 | 363.8 | 2.73 | 396.84 |
| Da2 | 6293 | 356 | 356.8 | 2.48 | 403.50 |
| Da3 | 7258 | 360 | 361.1 | 2.45 | 403.36 |
| Db1 | 4696 | 257 | 258.7 | 2.72 | 287.41 |
| Db2 | 5015 | 260 | 260.8 | 2.63 | 279.04 |
| Db3 | 5216 | 293 | 294 | 2.63 | 343.03 |
| Dc1 | 3437 | 282 | 283.5 | 3.46 | 313.47 |
| Dc2 | 3330 | 296 | 297.9 | 3.68 | 338.73 |
| Dc3 | 3343 | 285 | 285.8 | 3.5 | 315.11 |
| La1 | 5352 | 259 | 261.3 | 2.79 | 319.41 |
| La2 | 5372 | 260 | 261.2 | 2.74 | 317.72 |
| La3 | 3997 | 246 | 248.4 | 2.92 | 335.77 |
| Lb1 | 4536 | 279 | 280.6 | 2.86 | 320.03 |
| Lb2 | 4510 | 269 | 270.2 | 2.84 | 297.25 |
| Lb3 | 4874 | 282 | 283.3 | 2.84 | 320.26 |
| Lc1 | 5577 | 283 | 287.8 | 2.95 | 326.72 |
| Lc2 | 5742 | 295 | 297.1 | 2.83 | 341.19 |
| Lc3 | 5402 | 299 | 300.2 | 2.99 | 344.23 |
| Ma1 | 5066 | 300 | 301.3 | 2.91 | 363.62 |
| Ma2 | 5601 | 320 | 321.2 | 2.82 | 384.11 |
| Ma3 | 5539 | 281 | 282.3 | 2.74 | 327.08 |
| Mb1 | 5592 | 324 | 324.5 | 2.92 | 354.57 |
| Mb2 | 5687 | 305 | 305.5 | 2.62 | 335.35 |
| Mb3 | 5905 | 316 | 317.8 | 2.59 | 355.77 |
| Mc1 | 4392 | 278 | 280.1 | 3.28 | 317.32 |
| Mc2 | 4305 | 253 | 254.8 | 3.12 | 283.09 |
| Mc3 | 4412 | 265 | 266.5 | 3.11 | 309.47 |
| Wa1 | 4879 | 264 | 265.6 | 2.73 | 325.46 |
| Wa2 | 5013 | 252 | 253 | 2.63 | 306.99 |
| Wa3 | 5004 | 256 | 257.5 | 2.6 | 321.40 |
| Wb1 | 4767 | 298 | 301.4 | 3.33 | 348.26 |
| Wb2 | 4152 | 273 | 273.9 | 2.93 | 310.86 |
| Wb3 | 5105 | 325 | 326.8 | 3.19 | 379.74 |
| Wc1 | 4641 | 243 | 246.7 | 2.07 | 318.39 |
| Wc2 | 4726 | 237 | 237.7 | 1.99 | 268.99 |
| Wc3 | 4396 | 214 | 215.2 | 1.91 | 252.56 |
